# On the functional diversity of dynamical behaviour in genetic and metabolic feedback systems

**DOI:** 10.1186/1752-0509-3-51

**Published:** 2009-05-11

**Authors:** Lan K Nguyen, Don Kulasiri

**Affiliations:** 1 Centre for Advanced Computational Solutions (C-fACS), Lincoln University, Christchurch, New Zealand

## Abstract

**Background:**

Feedback regulation plays crucial roles in the robust control and maintenance of many cellular systems. Negative feedbacks are found to underline both stable and unstable, often oscillatory, behaviours. We explore the dynamical characteristics of systems with single as well as coupled negative feedback loops using a combined approach of analytical and numerical techniques. Particularly, we emphasise how the loop's characterising factors (strength and cooperativity levels) affect system dynamics and how individual loops interact in the coupled-loop systems.

**Results:**

We develop an analytical bifurcation analysis based on the stability and the Routh- Hurwitz theorem for a common negative feedback system and a variety of its variants. We demonstrate that different combinations of the feedback strengths of individual loops give rise to different dynamical behaviours. Moreover, incorporating more negative feedback loops always tend to enhance system stability. We show that two mechanisms, in addition to the lengthening of pathway, can lower the Hill coefficient to a biologically plausible level required for sustained oscillations. These include loops coupling and end-product utilisation. We find that the degradation rates solely affect the threshold Hill coefficient for sustained oscillation, while the synthesis rates have more significant roles in determining the threshold feedback strength. Unbalancing the degradation rates between the system species is found as a way to improve stability.

**Conclusion:**

The analytical methods and insights presented in this study demonstrate that reallocation of the feedback loop may or may not make the system more stable; the specific effect is determined by the degradation rates of the newly inhibited molecular species. As the loop moves closer to the end of the pathway, the minimum Hill coefficient for oscillation is reduced. Furthermore, under general (unequal) values of the degradation rates, system extension becomes more stable only when the added species degrades slower than it is being produced; otherwise the system is more prone to oscillation. The coupling of loops significantly increases the richness of dynamical bifurcation characteristics. The likelihood of having oscillatory behaviour is directly determined by the loops' strength: stronger loops always result in smaller oscillatory regions.

## Background

Feedback control mechanisms are vital to the robust functioning of gene regulatory and metabolic pathways. They have been extensively researched over the last two decades: we now know more about the topology and functionality of positive and negative feedback in intra- and inter-cellular systems than ever before [1]. For example, positive feedback is essential for the existence of multiple steady states within multi-scale gene regulatory systems [2] and to help prolong and amplify the response to weak signals in intracellular signalling [3]. Operating synchronously, negative feedback is found to help (1) stabilize and maintain the concentration of gene products, (2) maintain the homeostasis of gene expression rates [4], (3) improve the robustness of developing cells [3], and (4) facilitate the sustaining of oscillations of gene transcription rate [2]. Many examples of negative feedback systems exist: (1) regulatory pathways: the main repressor of the SOS regulon in bacteria *Escherichia coli*, *LexA*, represses its own production [5]; the Hes1 oscillator represses its own transcription [6]; the p53-Mdm2 network with p53 activates the *Mdm2 *gene and Mdm2 sequesters p53 [7]; and the tryptophan operon system with multiple negative feedback regulations [8]; (2) metabolic pathways: in the linear mandelate-acetate pathway in *Pseudomonas fluorescens *the acetate represses seven preceding reactions [9,10]; and, (3) signaling pathways: the NF-kB signalling pathway in which nuclear NF-kB activates production of IkBα which in turn inhibits nuclear import of NF-kB by sequestering it in the cytoplasm [11]; and circadian clocks [12]. These feedback loops orchestrate the molecular fluxes in multi-scale manner so that the organisms survive and thrive in many different environments.

Early work on negative feedback in gene regulatory systems goes back to that of Goodwin [13] who proposed an auto-repressive transcriptional model with inhibition imposed by the gene's own protein product. This initial model has provided a useful framework for later studies of systems involving negative feedback regulation [14-19]. In addition, a number of Goodwin-based models of biological oscillators characterised by one or more negative feedback loops have recently been developed, for example of the circadian clocks [20,21]. Nevertheless, our understanding of the dynamic nature of negative feedback regulation is still limited in many aspects. First, the mere presence of a negative feedback loop within a system is insufficient to understand its dynamical behaviour [22,23]. In fact, negative feedback is found to promote both system stability and oscillatory instability; the same feedback, if it is "loose", it may support stability, and if tighter, it may give rise to sustained oscillations [24]. Therefore, to gain deeper insights into the dynamical behaviours of biologically regulated systems, it is necessary for us to understand and characterize the differences among the feedback loops that influence system dynamics by systematically studying the different types of negative feedback loops that occur in these systems.

Two important factors that can be used to characterise a negative feedback loop are the feedback strength and level of binding cooperativity (nonlinearity) between an inhibitor and its regulated molecule [25,26]. In this paper we investigate the effects of these factors on system dynamics. Earlier work has only looked at the effects of changes in the cooperativity levels, but not those of feedback strength [14-19]. To coordinate complex and rich interactions within the cell, cellular systems often consist of not just one negative feedback loop, but multiple ones, entangled together. What are the functional advantages of the coupled feedback loops which evolved within the host systems? Although attempts have been made – e.g. the interplay between positive and negative feedback regulation have been shown to provide robustness and reliability to system performance [3,27] – the studies on how the coupled loops affect the molecular dynamics have been very limited. We therefore aim to explore in this paper the dynamical aspects of systems with multiple negative feedback loops in comparison with their single-loop counterparts to understand the possible functional advantages the extra loops may provide.

We consider a commonly encountered motif of the negative feedback systems in which negative feedback is imposed by the last species of the system pathway on the upstream species. We develop mathematical models to analyze for stability and bifurcation, in order to study the behaviour of these systems, confining ourselves to the analytical solutions which allow us to obtain bifurcation points dependent on the feedback strengths and nonlinearity as the parameters. Based on these results, the conditions on the feedback strength or nonlinearity for: no stability; no oscillation; stability enhancement; oscillation enhancement; and guaranteed stability (oscillation) can be established. Our analyses will lead to regimes in the parameter space in which different dynamical behaviours can be identified. In contrast to numerical methods, analytical methods facilitate an analysis of the parameter sensitivities of the system dynamics.

For clarification, here we define the meanings of *robustness *and *stability *used in this paper. Robustness is referred to as the ability of a system to maintain its functionality against internal perturbations and environmental variations. Stability, on the other hand, is only concerned with the ability to maintain the system state. Although both are important properties of living systems, robustness is a broader concept than stability, with the emphasis on system functionality rather than system state [28]. A system can preserve its function amid perturbations by actively switching between different (stable and unstable) states [28]. In this study, we focused on the stability aspect of systems.

The remaining structure of this paper is given as follows: the Methods section discusses Hill function and its use for negative feedback modelling, followed by a description of the regulatory motifs studied in the current paper and their mathematical models. The analytical methods developed for stability and bifurcation analyses are outlined in the last subsection. Detailed analyses along with discussion of pertinent results are presented in the section Results and Discussion. In the section Biological Examples, we analyze the Hes1 oscillator in vertebrates and the Tryptophan operon system in bacteria *Escherichia coli *as examples. Finally, we end the paper with the Summary and Conclusion section.

## Methods

### Modelling negative feedbacks – Hill function

In modelling biochemical systems, the rate of a reaction representing concentration change per unit time can be written as a function of the concentrations of reactants and products. There exist a number of rate laws corresponding to different types of reaction mechanisms: the mass action rate law, the Michaelis-Menten kinetics, and the Hill functions [1,29]. The level of inhibition caused by negative feedback loop due to product *X *can be described by the Hill function of the following form (another form of the Hill function can also be used to model activation – [1]):



where the parameter *K*_i _represents the half-saturation constant (i.e. the concentration of *X *that gives 0.5 ratio repression). It is also commonly referred to as the dissociation constant or binding constant. The parameter *n *(Hill coefficient) is related to the cooperativity level of the chemical process.

**Figure 1:**
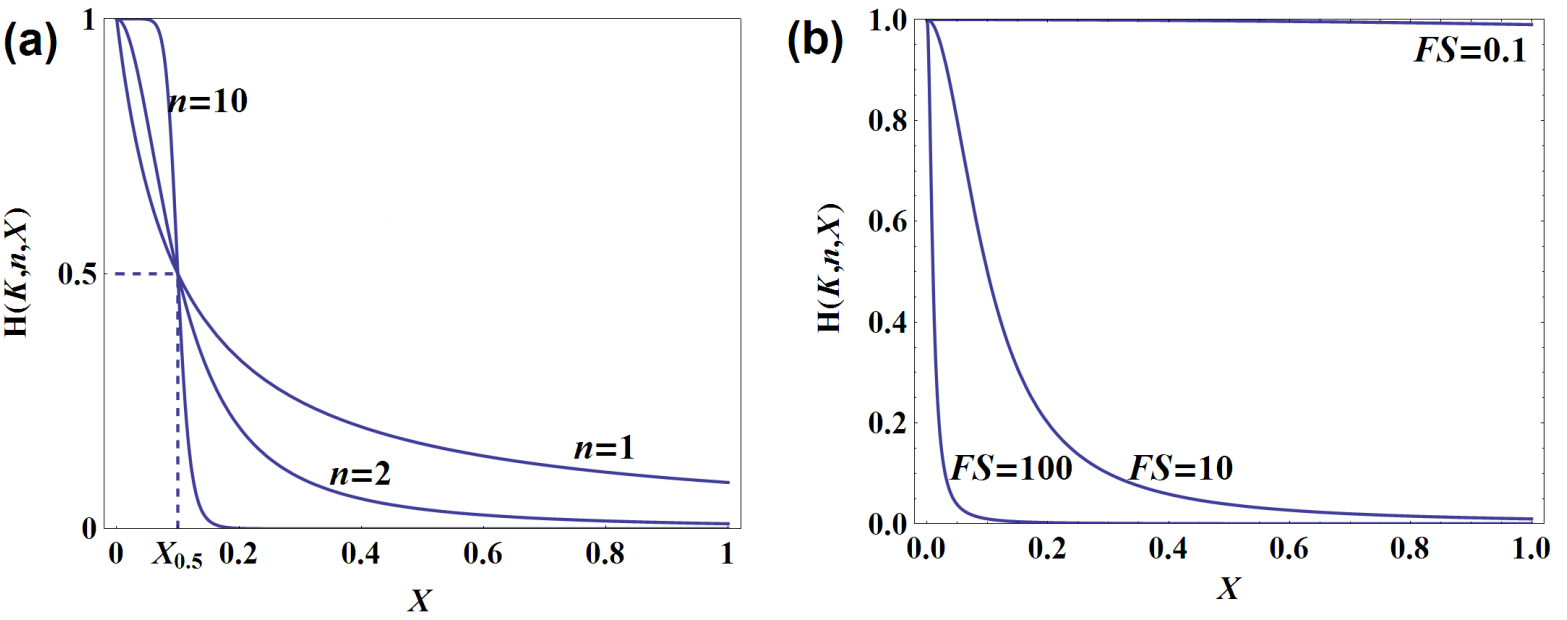
**Hill function for modelling inhibition**. (a) Hill function with increasing *n *(FS is fixed at 10) (b) Hill function with decreasing FS (*n *is fixed at 2).

The strength of feedback is inversely proportional to *K*_i_: increasing *K*_i _lowers the repression level while decreasing *K*_i _increases the repression for a given *n*; therefore we may define **FS = 1/*K *as feedback strength **(Figure 1); and refer to *K *as the **inverse feedback strength indicator**. The Hill coefficient *n *can be interpreted as the sensitivity of the feedback loop. As *n *becomes larger, the Hill curve becomes more sensitive to change in *X *in the vicinity of *X*_0.5 _and acts like an on-off switch. When *n *is very large (infinity), the Hill function resembles the step function (Figure 1).

In gene regulatory networks, change in FS could be brought about in a number of ways: (1) by mutations that alter the DNA sequence of the binding site of *X *in the inhibited molecular species' promoter – even alternation of a single DNA base can strengthen or weaken the chemical bonds between *X *and the DNA – which will subsequently change FS; (2) by change of binding site position within the DNA. The Hill coefficient can be changed, for example, by mutations that alter number of binding sites within the DNA. It has been experimentally shown for bacteria that they can accurately tune these parameters within only several hundred generations for optimal performance when faced with environmental change [1].

### Regulatory Motifs and Models

**Figure 2:**
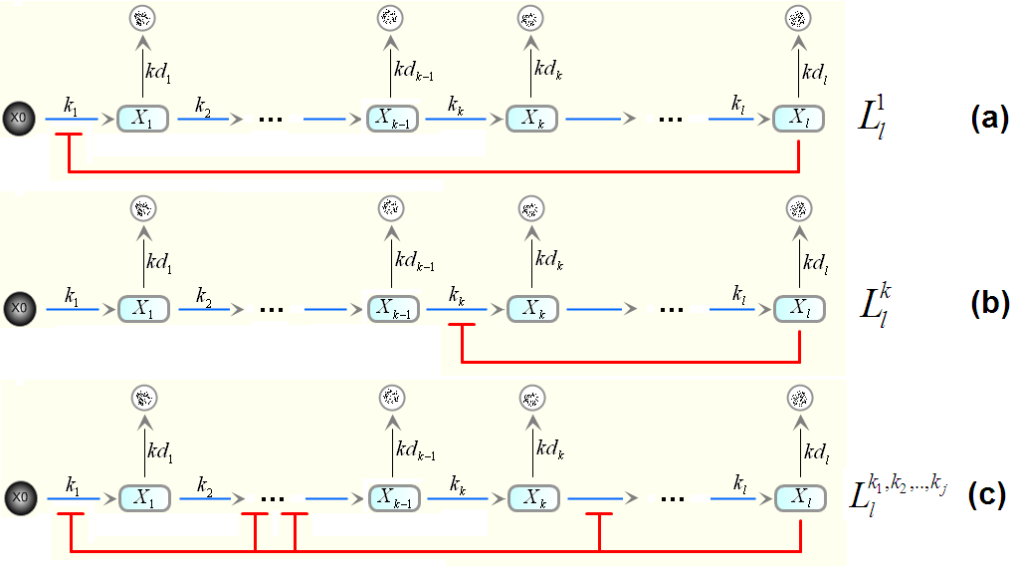
**Schematic network structures of example negative feedback systems**. **(a) **The classical Goodwin system with length *l*. **(b) **The modified Goodwin system with the feedback loop reallocated to repress step *k*th reaction. **(c) **The multiple-loop system.

Our model motifs consist of generic pathways of activation steps (reactions) with arbitrary lengths with single or coupled negative feedback loops imposed by the end-product of the pathway. Figure 2 shows the schematic diagrams of a few example motifs. We denote a general *l*-species system with length *l *by *L*_*l*_; while a system having single feedback loop on the step *k *is denoted by  (1 ≤ *k *≤ *l*). The system with the coupled loops on the steps *k*_1_, *k*_2_,.., *k*_*j *_is denoted by .

A model system can be described by using a set of *l *differential equations as follows,



where *x*_*j*_, *k*_*j*_, *k*_*dj *_(*j *= 1,.., *l*) represent the concentration of species *X*_j_, its synthesis rate, and its degradation rate, respectively. And,

 if feedback loop is present while

*H*(*K*_*j*_, *n*_*j*_, *x*_*l*_) = 1 if no feedback loop is present for the j^th ^step.

The first species of the pathways *X*_0 _is often assumed to be static, i.e. its concentration is unchanged. In most cases, it represents the gene which is the source of the pathway and activates the whole sequence of reactions. However, we also consider the cases when *X*_0 _is variable by studying the variants of system *L*_*l *_with shorter lengths. The degradation process of model species is assumed to follow first-order kinetics. Degradation parameter *k*_dj _is actually an aggregated rate combining transport (or modification) and decay rate of corresponding species. The Goodwin oscillator is a special case and represented by system . We do not consider time delay in these models; readers who are interested in this aspect are referred to the work of MacDonald [30].

Despite their simplicity, these models can readily be used to model real biological systems. For instance, a generic model with three variables can be interpreted as follows: the first equation as the synthesis of nucleic mRNA, the second equation describing transportation of mRNA to the cytoplasm, while the last equation explains translation of mRNA into protein. Extended four-variable system can be interpreted by including a fourth equation describing transportation of protein back to the nucleus.

We first analyse the single-loop systems with three and four variables. The coupled-loops systems are examined next. We then study the generalised, extended systems with arbitrary pathway lengths.

### Analysis Methodology

Biological systems display many types of dynamic behaviours including stable steady state, sustained oscillations, and irregular fluctuating dynamics (chaos). Change of system parameters may lead to change of system dynamics. Bifurcation analysis allows one to subdivide the parameter space into qualitatively different regions within each, the system dynamics are homogeneous. Furthermore, the changes in the size and location of resulting regions due to parameters variation can be investigated.

#### A Summary of Stability Analysis and the Routh-Hurwitz Theorem

The stability analysis of a system consisting of a set of differential equations can be conducted by considering its dynamical behaviour in the neighbourhood of its equilibrium (i.e. steady) state. A steady state is classified locally stable if the system returns to this steady state after a sufficiently small but arbitrary perturbation. Local stability of a steady state can be analysed by linearising the differential equations around the steady state and assessing the eigenvalues of the resulting Jacobian matrix (*J*) [31]. For a system of differential equations



If the real parts of all *J*'s eigenvalues are negative, the steady state is said to be stable, while if any of the real parts are positive, the steady state is unstable (in this case the system oscillates if the imaginary part is nonzero).

Because *J*'s eigenvalues are actually the roots of the following characteristic equation



- *α*_i_s are the coefficients – to assess the signs of *J*' eigenvalues, we make use of the Routh-Hurwitz theorem [31,32] which states that eigenvalues *λ *all have negative real parts if



where



#### Bifurcation – a Geometrically Motivated Approach

**Figure 3:**
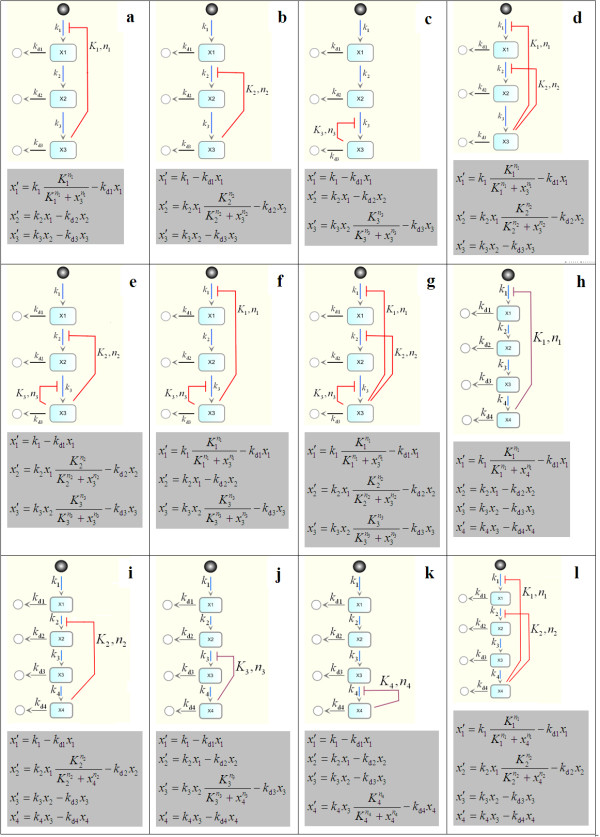
**Schematic diagram of feedback motifs analysed in the paper together with their model equations**.

Our aim is to establish analytical bifurcation points for the feedback strengths, Hill coefficients, and other model parameters of single-loop as well as multiple-loop systems. System stability conditions are first formulated using the Routh-Hurwitz stability criteria outlined above. These conditions are then examined using a geometrically-motivated approach. We demonstrate the method below using a system with length 3. Longer pathway systems are similarly analysed. Consider the case where all three loops are present (Figure 3g), and the equations for this system are:

(1)

Denote the equilibrium values of the state variables *x*_i_, (*i *= 1, 2, 3). Steady-state (equilibrium) values of the system variables can be determined by setting the right hand sides of (1) to zeros. This subsequently gives (see Additional file 1)

(2)

where



In this case, the characteristic polynomial is cubic



Following the Routh-Hurwitz theorem, the system is stable if and only if the following Stability Condition (SC) is satisfied:

(3)

It is convenient to introduce the following composite variables

(4)

Variables *M*_1_, *M*_2_, *M*_3 _interestingly have the characteristics of activation functions. Working with *M*_1_, *M*_2_, *M*_3 _is more straightforward than with *x*_1_, *x*_2 _and *x*_3 _directly as it spares one from having to deal with the exponential and rational forms in (2). We also have 0 <*M*_1_, *M*_2_, *M*_3 _< 1. The equilibrium condition (2) is now simplified to

(5)

Equations (4) also allow the characteristic coefficients *α*_1_, *α*_2 _and *α*_3 _to be expressed in terms of only *M*_1_, *M*_2_, *M*_3 _and other model parameters, i.e. the synthetic and degradation rates (see equations (9) below for example). Particularly, the conditions (3) and (5) for simpler system motifs with less feedback loops can be easily derived. For example, setting *M*_i _= 0 for some index *i *gives rise to a system structure lacking the corresponding feedback loop, e.g. *M*_1 _= 0 gives , *M*_1 _= *M*_2 _= 0 gives . Equations (4) lead to:

(6)

Combined with (5) and (6), each of *M*_1_, *M*_2 _and *M*_3 _can be expressed as functions of the others involving only *K*_i_s and *n*_i_s. For example, assume *M*_3 _= 0 for simplicity (system ), we have



Here, *M*_1 _is a strictly decreasing function of *M*_2 _over (0, 1). Substitute this into , we obtain:

(7)

as a function of *K*_2 _and *M*_2_. Moreover, *K*_1 _is strictly increasing with *M*_2 _since the derivatives with respect to *M*_2 _of the terms inside the brackets in (7) are positive over (0, 1) (see section 4.1 in Additional file 1).

This means if there exist bounds *M*_2l_, *M*_2h _(based on equation (3)) such that *M*_2l _≤ *M*_2 _≤ *M*_2h_, then the stability condition (3) would be equivalent to

(8)

Condition (8) represents an analytical relationship between the feedback strength indicators *K*_1 _and *K*_2 _of the loops in action, and *f*_1_(*M*_2l_, *K*_2_) and *f*_1_(*M*_2h_, *K*_2_) are the bifurcation points of *K*_1_.

To determine the bounds *M*_2l _and *M*_2h_, note that (3) can be manipulated to take the form



where *g *is a function whose explicit form depends on the particular system motif. For system  we have

(9)

Because *α*_1_, *α*_2_, *α*_3 _> 0, (3) is equivalent to *α*_1_*α*_2 _- *α*_3 _> 0. Substituting (9) into this relation we obtain

(10)

where *f*(*M*_2_) = *M*_1 _and , with coefficients *a*_0_, *a*_1 _and *b*_1 _are expressions of the system parameters and given in the supplementary material (section 4.2, Additional file 1).

**Figure 4:**
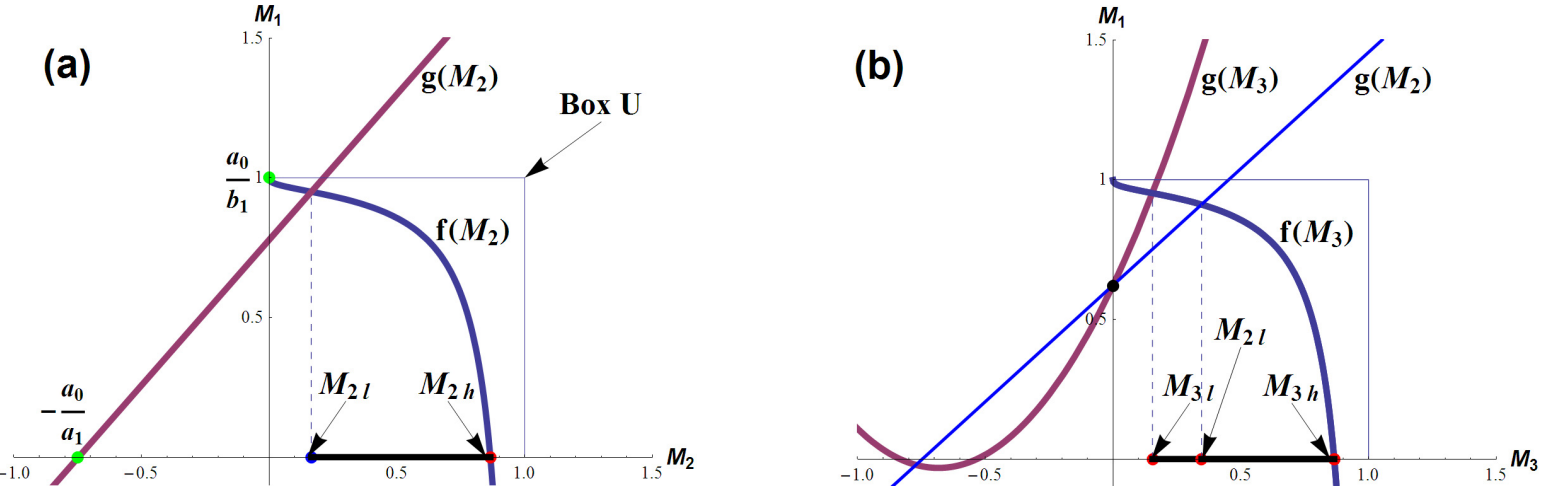
**(a) Functions *f*(*M*_2_) and *g*(*M*_2_) on the two-dimensional *M*_1_-*M*_2 _coordinate**. Lower (*M*_2l_) and upper (*M*_2h_) bounds of *M*_2 _satisfying the stability condition are indicated on the *M*_2 _axes (the figure is produced with parameter values: *n*_1 _= 12, *n*_2 _= 3, *k*_1 _= 1, *k*_2 _= 2, *k*_3 _= 1, *k*_d1 _= 1.2, *k*_d2 _= 3, *k*_d3 _= 2 and *K*_2 _= 0.02). **(b)** Determining curve *f*(*M*_3_) (solid blue) and *g*(*M*_3_) (dashed purple) on the two-dimensional *M*_1_-*M*_3 _coordinate.

Note that (10) is to take different forms depending on specific motifs of feedback loops. Next, we analyse the inequality (10) for the system  using a geometrical approach in which the curves *f*(*M*_2_) and *g*(*M*_2_) are drawn on the two dimensional *M*_1_-*M*_2 _coordinate plane (Figure 4a). *f*(*M*_2_) is a strictly decreasing curve contained within the unit square U = {(0,0); (1,0); (1,1); (0,1)} (indicated with dashed boundary in Figure 4a), *g*(*M*_2_) is a straight line with a positive slope. As 0 <*M*_1_, *M*_2 _< 1, the analysis is constrained within U only. Range of *M*_2 _satisfying (10) can be determined along with its lower and upper bounds *M*_2l _and *M*_2h_, illustrated in Figure 4a.

## Results and discussion

### Single-loop Systems

Because two-species systems are incapable of demonstrating oscillatory dynamics, we only consider systems with three species or more. Here, we present the results for the systems with a single feedback loop. To this end, the three-species systems are first considered. We then examine the four-species systems and investigate potential effects on system functional dynamics as a result of lengthening the pathways.

#### Three-species Systems

Three negative feedback motifs are possible for the three-species system where the feedback loop is imposed on the first, second, and the last step of the pathway. These are denoted ,  and  and schematically demonstrated in Figure 3a, b, and 3c, respectively. We found that systems  and  are both incapable of having oscillatory dynamics, regardless of their parameter values. System , essentially the Goodwin system of length three, possesses both stable and oscillatory dynamics. Switching between these dynamical regimes occurs through a Hopf bifurcation. We present below the analytical condition governing this bifurcation.

The system is stable if and only if the following condition is satisfied (see section 1.1.1 in Additional file 1 for the derivation):

(11)

where

(12)

##### K_1 _versus n_1_

Manipulating the inequality (11) yields an equivalent condition between the inverse feedback strength indicator *K*_1 _versus the Hill coefficient *n*_1 _and the remaining model parameters (i.e. the synthetic and degradation rates – see section 1.1.4 in Additional file 1 for the derivation), given below

(13)

where

(14)

**Figure 5:**
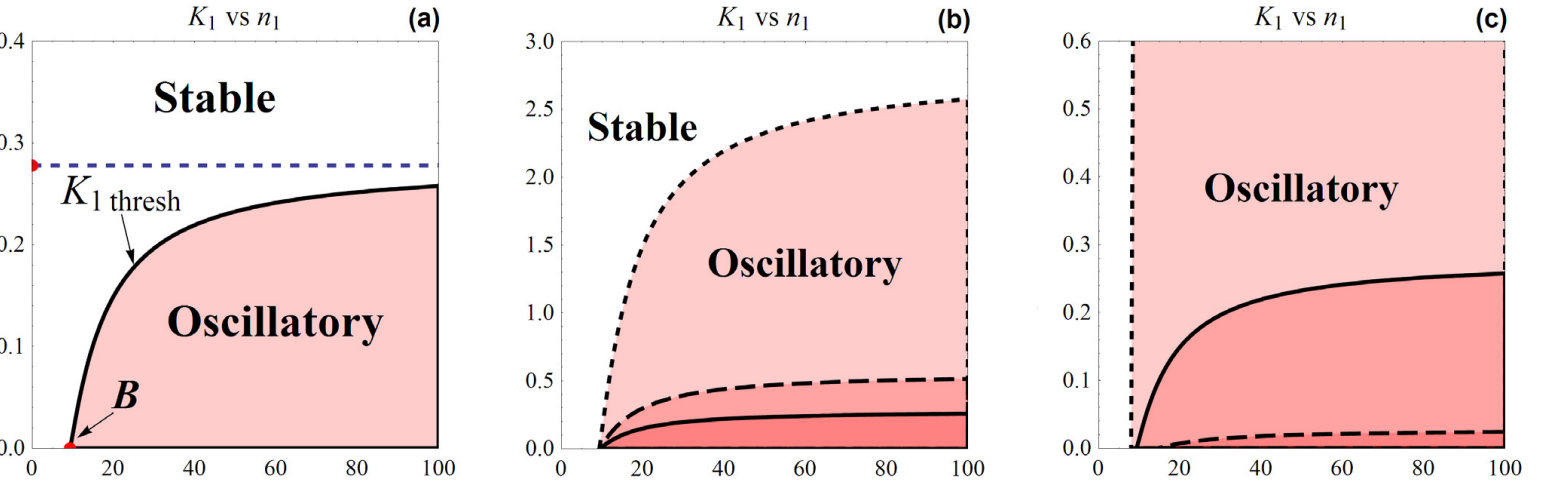
**(a) Bifurcation diagram of *K*_1 _against *n*_1_**. The stable and oscillatory regions are separated by the *K*_1thesh _curve which increasingly approaches the *K*_1crit _line (dashed). *B*, indicated on the *n*_1_-axis, is the minimum value of the Hill coefficient at which oscillations are possible (parameter values *k*_1 _= 1, *k*_2 _= 2, *k*_3 _= 1, *k*_d1 _= 1.2, *k*_d2 _= 3, *k*_d3 _= 2 were used for graphing). **(b, c) **Comparison of the *K*_1 _vs. *n*_1 _bifurcation diagrams for different scenarios.** (b) ***K*_1 _vs. *n*_1 _bifurcation diagram for the base parameter set, *k*_1 _= 1, *k*_2 _= 2, *k*_3 _= 1, *k*_d1 _= 1.2, *k*_d2 _= 3, *k*_d3 _= 2, (solid); when a synthesis rate *k*_1 _is doubled (dashed); and 10-times increased (dot). Note that the exact same effects are also obtained for changing other synthesis rates. **(c) ***K*_1 _vs. *n*_1 _bifurcation diagram for the base parameter set (solid) compared to the set in which the degradation rate *k*_d1 _is increased 10 times (dashed), and the set when the degradation rates are set identical, *k*_d1 _= *k*_d2 _= *k*_d3 _= 0.5 (dot).

This shows, given other model parameters' values, the existence of a threshold feedback strength (1/*K*_1thresh_) at which the system loses stability to an oscillatory regime. Based on (13), two-parameter bifurcation diagrams of *K*_1 _against other model parameters can be set up. Figure 5a illustrates on the *K*_1 _vs. *n*_1 _plane, regions of stable and oscillatory dynamics, separated by the *K*_1thresh _curve.

To obtain oscillatory behaviour, the feedback loop must be sufficiently strong (*K*_1 _below the curve). Furthermore, *K*_1thresh _saturates at high *n*_1 _which indicates that there exists a critical value of *K*_1 _or feedback strength above which, the system is guaranteed to have sustained oscillations regardless of the value of the Hill coefficient *n*_1 _(Figure 5a). Denote this critical value *K*_1crit_, we found that *K*_1crit _= 1/*A*, which is the product of the system synthesis rates divided by the product of the system degradation rates (see section 1.1.5 in Additional file 1 for the derivation). Figure 5a further shows that higher cooperativity level improves the likelihood to observe oscillation, since oscillation is obtained over a wider range of *K*_1_, i.e. lower *n*_1 _provides more stability. However, this improvement diminishes at high cooperativity level due to the saturation behaviour of *K*_1thresh_.

##### Parameter's "Ranges of Guaranteed Stability"

Here, we define the Ranges of Guaranteed Stability (RGS) of a model parameter *p *with respect to model parameter *q *as all possible values of *p *that always give a stable system dynamics, subjected to arbitrary variation in *q*. For instance, as shown above, *K*_1 _> 1/*A *or (1/*A*, +∞) is the RGS of *K*_1 _with respect to *n*_1_.

Because *M*_1 _< 1, equation (11) means that the system is always stable if *n*_1 _≤ *B*. This threshold value depends on the degradation rates only. It also yields the RGS of *n*_1 _to equal (0, *B*], with respect to all model parameters except the degradation rates. Since *B *≥ 8 for any arbitrary values of *k*_d1_, *k*_d2 _and *k*_d3_, the interval (0,8] becomes the "global" RGS of *n*_1_, i.e. with respect to all model parameters. Furthermore, for any *n*_1 _≥ 8, the system can be made oscillating with a proper set of the degradation rates and having a sufficiently weak feedback loop. The number of these sets is found indefinite and shown in section 1.1.3 in Additional file 1.

##### Effects of turnover parameters

Here, we investigate effect of the synthesis and degradation parameters on the system's bifurcation characteristics. Since *A *and *B *are symmetrical expressions, *K*_1thresh _is also symmetrical with respect to the degradation as well as the synthesis rates. This means that all system species equally affect the system's bifurcation characteristics in spite of the fact that the feedback loop is only acting on the first reaction of the pathway.

Regarding the synthesis rates, *K*_1thresh _changes proportionally with these parameters. Increase in the production of any of the model species therefore gives rise to a more oscillatory-prone system, indicated by a larger oscillatory region in the two-parameter *K*_1 _vs. *n*_1 _plane (Figure 5b). More interestingly, raising the production rate of any species results in an exactly same *K*_1thresh _curve as raising the production rate of any other species by the same proportion. Bifurcation patterns are therefore conserved under these different changes. This knowledge is potentially useful in many cases. For example, it can facilitate the engineering of synthetic circuits with desirable dynamical behaviour; as one could effectively choose appropriate points to perturb to attain desired dynamical behaviours. It can also help in the process of parameter estimation and optimisation of synthetic circuits.

The degradation rates, on the other hand, have opposite effect on system dynamics. Higher degradation rates tend to reduce *K*_1thresh_, leading to smaller oscillatory region (Figure 5c) and consequently a more stable system. System stability, therefore, is most likely when model species are rapidly degraded. This is because at large *k*_dj_, rapid degradations of the state species significantly weaken the strength of the negative feedback loop that is required for oscillations. In contrast, very slow degradation makes it almost impossible for the system to obtain stability, unless the feedback loop is greatly relaxed with significantly weak inhibition strength (i.e. very high *K*_1_).

In examining parameter effects on the threshold value of the Hill coefficient (*B*), our analysis reveals that comparable degradation rates across model species (*k*_d1 _≈ *k*_d2 _≈ *k*_d3_) leads to minimum *B *and thus minimum RGS for *n*_1_; whereas if one is many folds greater than another (*k*_di _>> *k*_dj_, i, j ∈ {1, 2, 3}), *B *will be high, resulting in a large RGS (see section 1.1.6 in Additional file 1 for the justification). This suggests a way to enhance system stability by unbalancing the degradation rates of molecular species, preferably, towards higher values. Figure 5c compares the bifurcation profiles between a reference parameter set and one with equally low degradation rates; and one with unequal, enhanced degradation rates.

#### Four-species Systems

These systems can be considered extension of the previous models, which consist of four species. There are a total of four feedback motifs for single-loop systems. Similar to  and , systems  and  are found to be incapable of producing oscillations. Here, we consider  and  in turn (Figure 3h, i).

Interestingly for , our analysis arrives at the same bifurcation points for *n*_1 _and *K*_1 _as in (11) and (13), however with different expressions of *A *and *B*:

(15)

(16)

We found that *B *≥ 4 for arbitrary values of the degradation rates. Compared with the three-species system, *n*_1_'s RGS is reduced, supposedly due to the lengthening of system pathway. Furthermore, for *n*_1 _> 4 the system is capable of displaying sustained oscillation for an indefinite number of parameter sets, given a proper selection (see section 1.2 in Additional file 1 for the justification).

Let us now consider system , a variant of the single-loop Goodwin system where inhibition is imposed by the end-product on the second rather than the first step of the pathway (Figure 3i). At first glance, this system design looks like its counterpart . However, there is a fundamental difference in the synthesis of the repressed variables between the two systems. The synthetic term for *x*_2 _in  contains *x*_1 _which changes dynamically, having its rate of change defined by the first model equation in Figure 3i; whereas the synthetic term for *x*_1 _in  does not contain a varying variable;  can thus be considered as a special case of  by setting *k*_d1 _= 0 in Figure 3i. This difference might give rise to distinct dynamical behaviours between the two systems. Here, we analyse 's bifurcation to identify and investigate possible behavioural discrepancies compared to .

For system , the analytical bifurcation point for feedback strength *K*_2 _is



where *A *has the form of (15) while *B *is reduced to resemble that of the three-species system  in equation (12),

(17)

#### Generalised Single-loop Systems

##### Minimum Hill Coefficient for Sustained Oscillation

In fact, for the single-loop Goodwin system with arbitrary length *l*, the minimum Hill coefficient required for sustained oscillations has been theoretically computed to be



although this calculation is done under the stringent assumption of equal degradation rates *k*_d1 _= *k*_d2 _=...= *k*_*dl *_[15,19].

**Figure 6:**
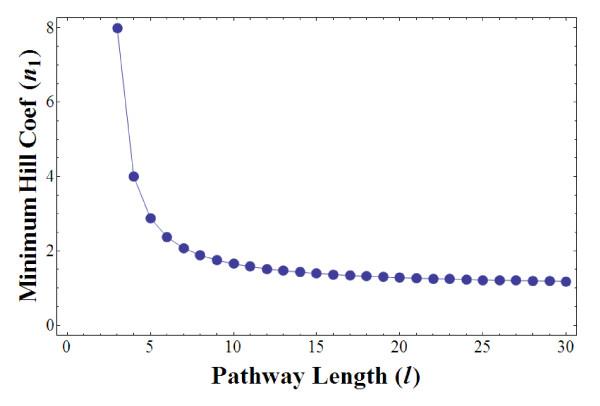
**Minimum Hill coefficient (*n*_1_) required for sustained oscillations in the Goodwin system with length *l***.

Figure 6 plots this minimum Hill coefficient against the pathway length *l *(≥ 3). We observe dramatic reduction of the minimum *n*_1 _at small length (≤ 10) but this reduction becomes insignificant for longer pathway; a saturation trend is instead observed. Our derivation, however, gives us explicit form of the minimum *n*_1 _as analytical expression of the degradation rates.

##### Effects of System Extension

Comparative study of the systems  and  allows us to examine dynamical effects resulting from system extension (i.e. the lengthening of system pathway). We found that the extended system with more species is not always more stable. In fact, whether the extended system is more stable or more prone to oscillation is determined by the kinetics (i.e. synthetic and degradation rates) of the added species.

If we denote *K*_1crit_(*L*) the critical *K*_1 _value of system *L*, (14) and (15) then give

(18)

Equation (18) indicates that if the additional species is more quickly degraded than produced (*k*_4 _<*k*_d4_), *K*_1crit _will be reduced for the extended system. On the other hand, *K*_1crit _for  is raised if *k*_4 _> *k*_d4 _and unchanged if *k*_4 _= *k*_d4_. Also, note that if *k*_d4 _is small (large) relative to other degradation rates, *n*_1crit _for the extended system becomes greater (smaller) (see (12) and (16)).

**Figure 7:**
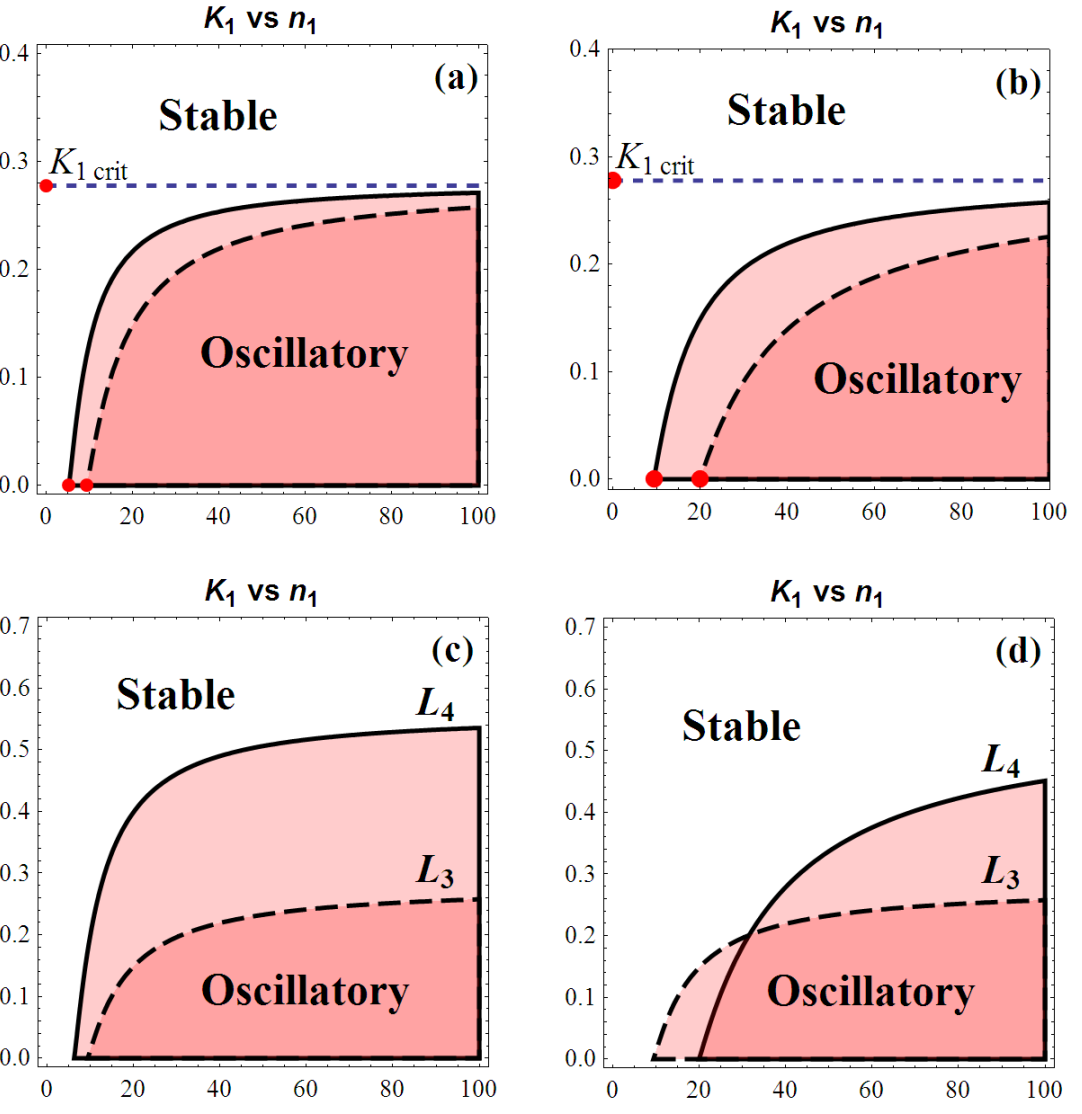
**Comparison of the two-parameter bifurcation diagram *K*_1 _vs. *n*_1 _between the 3 and 4-species systems: *L*_3 _(solid) and *L*_4 _(dashed)**. (a) *k*_d4 _= *k*_4 _for added species, *K*_1crit _is retained; (b)*k*_d4 _= *k*_4 _but small *k*_d4_, *K*_1crit _is retained but *L*_4 _has smaller oscillatory region; (c) *k*_d4 _= 0.5*k*_4_, *K*_1crit _is doubled, extended system has approximately doubled oscillatory region; (d) *k*_d4 _= 0.5*k*_4 _but with small *k*_d4_, *K*_1crit _is doubled, however *B*_1_> *B*, *L*_3 _and *L*_4 _have overlapping oscillatory regions. We used parameter sets (*k*_1 _= 1, *k*_2 _= 2, *k*_3 _= 1, *k*_d1 _= 1.2, *k*_d2 _= 3, *k*_d3 _= 2) and (*k*_4_, *k*_d4_) = (5,5) for (a); (0.1,0.1) for (b); (1,0.5) for (c) and (0.2, 0.1) for (d).

Figure 7 demonstrates the effects of system extension on the bifurcation characteristics under different scenarios of the added species' kinetics. We conclude that the extended system obtained as a consequence of pathway lengthening becomes more stable only when the added species degrades slower than it is being produced. In this case, the feedback loop must increase its strength to a proportional level, if sustained oscillation is to be obtained (see (18)). On the other hand, the extended systems are more prone to sustained oscillations if the additional species degrades faster and being created. These observations provide us with useful indications of how regulatory system might tune its feedback strength to achieve certain types of dynamics.

##### Generalisation of Feedback Strength and Hill coefficient

The equation defining the threshold feedback strength for the systems ,  and  can be extended by induction to a system with an arbitrary number of reaction steps. The threshold feedback strength of the Goodwin system with the general length *l *( – schematic diagram in Figure 2), for example, can be expressed as in equation (13) with *A*'s generalised form below,

(19)

and *B *is a function of only the degradation rates *k*_d1_, *k*_d2_,..., *k*_dl_. *B *was found for the systems with length 3 and 4 to be neat expressions of the degradation rates. However, for the system with 5 variables or more, *B *becomes complex but can be derived explicitly. Moreover, *B *reaches its minimum when all degradation rates are equal, at which B = 1/*Cos^l^*(*π/l*). In addition, the generalised critical value of the feedback strength is *A*. A system with feedback strength weaker than this value is guaranteed a stable dynamics independent of the Hill coefficient values.

**Figure 8:**
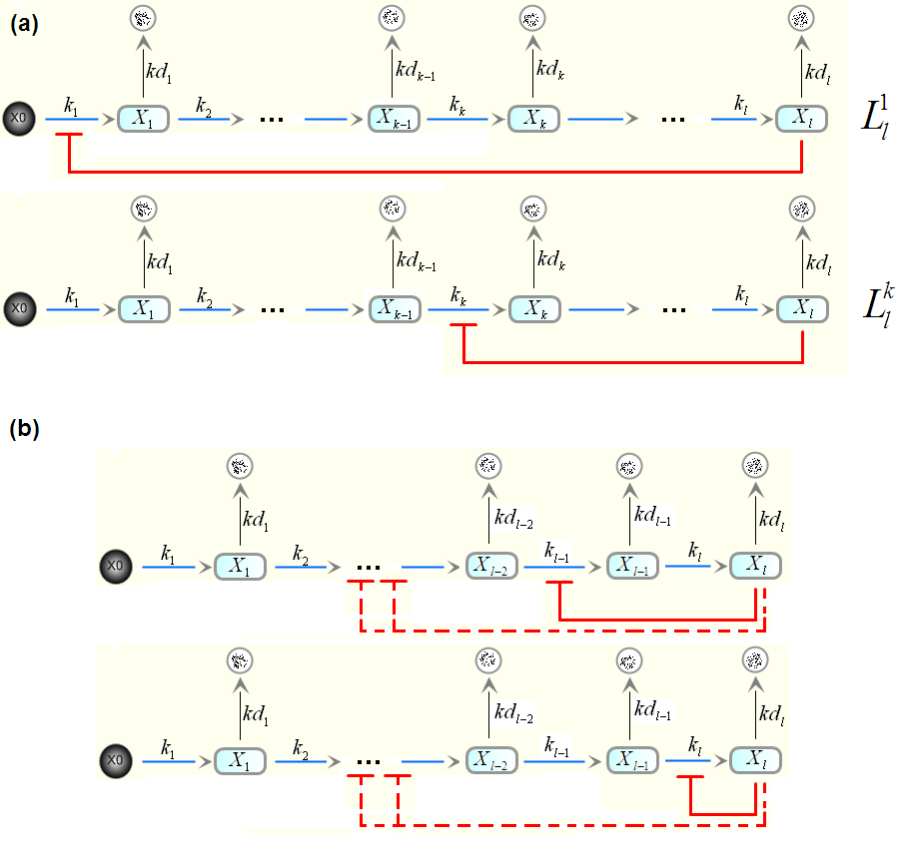
**(a) Schematic topology of the general Goodwin system () and its variants. **(b) Schematic diagram of non-oscillatory systems. These include single as well as multiple-loop systems consisting of at least one loop on the last or second-last pathway step.

Equation (17) shows that the threshold Hill coefficient of  only depends on the degradation rates of the inhibited species and its downstream molecules; and is independent of the upstream species. More interestingly, equations (12) and (17) show that  and  share the same analytical form for *B*. Further generalisations, therefore, can be made for variants of the general Goodwin systems: the systems with single negative feedback loop in which any step in the biochemical pathway can be potentially inhibited by the end-product (i.e. systems  with 1 <= *k *<= *I *- Figure 8a). The threshold *K*_kthresh _of  is

(20)

where *A *is retained its form as in (19) whereas *B *involves only the degradation rates of downstream species of the repressive targeted species *X*_*k*_.

More importantly, *B *has similar form as that of the reduced Goodwin system  (Figure S1 in Additional file 1). We confirmed these generalised analytical equations using numerical computations as well in which we estimate *K*_kthesh _for variant systems  and found that they fit the theoretical form given by .

##### Effects of Feedback Loop Reallocation

The generalised findings above have important implications concerning the dynamical behaviour of feedback systems. Comparing (19) and (20) reveals that reallocation of the negative feedback loop has no effect on the critical feedback strength (1/*K*_crit_). Regardless of the loop's position, the system's stability is ensured if the feedback strength is weaker than this value.

However, loop reallocation may or may not make the system more stable. This is because the threshold Hill coefficient values *B *in (19) and (20) are different. The ratios of *B *for  and  based on equations (16) and (17) is given by



For simplification, let *k*_d2 _= *k*_d3 _= *k*_d4 _= 1. We observe that *B*() >> *B*() when *k*_d1 _<< 1; *B*() <*B*() when *k*_d1 _≈ 1 but *B*() approaches *B*() when *k*_d1 _>> 1. This means *B *could become either larger or smaller depending on the relative magnitude of *k*_d1 _compared to *k*_d2_, *k*_d3_, *k*_d4_. Consequently, the oscillatory region on the two-dimensional bifurcation diagram *K*_1 _vs. *n*_1 _could either shrink or expand due to loop reallocation.

##### Non-oscillatory Systems

Because the systems with less than three species are not able to display oscillatory dynamics, the generalised results obtained in the previous section indicate that for the systems with arbitrary length which possess a single loop inhibiting either the last step or second-last step of the pathways ( or ), oscillatory dynamics is also not feasible. This conclusion is in line with analytical results obtained for the systems , , ,  previously.

We will show in the next section that, adding extra inhibition loops tends to make the system less likely to demonstrate sustained oscillations. This implies that any system with multiple negative feedback loops encompassing one imposing on either the last or second last step ( or  with *k*_1_,.., *k*_*m *_∈ {1, 2,.., *l*}) is also incapable of having oscillatory dynamics (Figure 8b).

### Coupled-loop Systems

Let us consider systems with increased complexity in which several negative feedback loops are coupled together. Understanding of composite behaviour of coupled loops has been limited. We aim to establish meaningful connections between the feedback strength of these loops (through the inverse indicator parameters *K*s) under certain conditions.

#### Three-species, Doubled-loop System 

Detailed analysis for this system was presented in section "Analysis Methodology" as an example (Figure 3d). The stability condition (10) is ensured if *g*(*M*_2_) intersect the *M*_1_-axis at a point above point (0,1), indicated by a dot in Figure 4a. This translates *a*_0 _≥ *b*_1 _or *n*_1 _≤ *B *with *B *as in (12). Compared to the results from the previous section of the three-species, single-loop system, we found that adding a second feedback loop does not affect the RGS of *n*_1_. On the other hand, if *n*_1 _> *B*, the line *g*(*M*_2_) must intersect *f*(*M*_2_) within the unit square U and so condition (10) is violated for some *M*_2_, subsequently destabilising the system (Figure 3a).

##### K_1_-K_2 _Bifurcation Diagram

Range of *M*_2 _satisfying (10) can be determined, indicated by its lower bound *M*_2l _and higher bound *M*_2h _in Figure 4a. For each value of *K*_2_, we obtain corresponding values for *M*_2l _and *M*_2h_. Using (8), we construct the two-parameter bifurcation diagram with the feedback strength indicators *K*_1 _and *K*_2 _being the axes.

**Figure 9:**
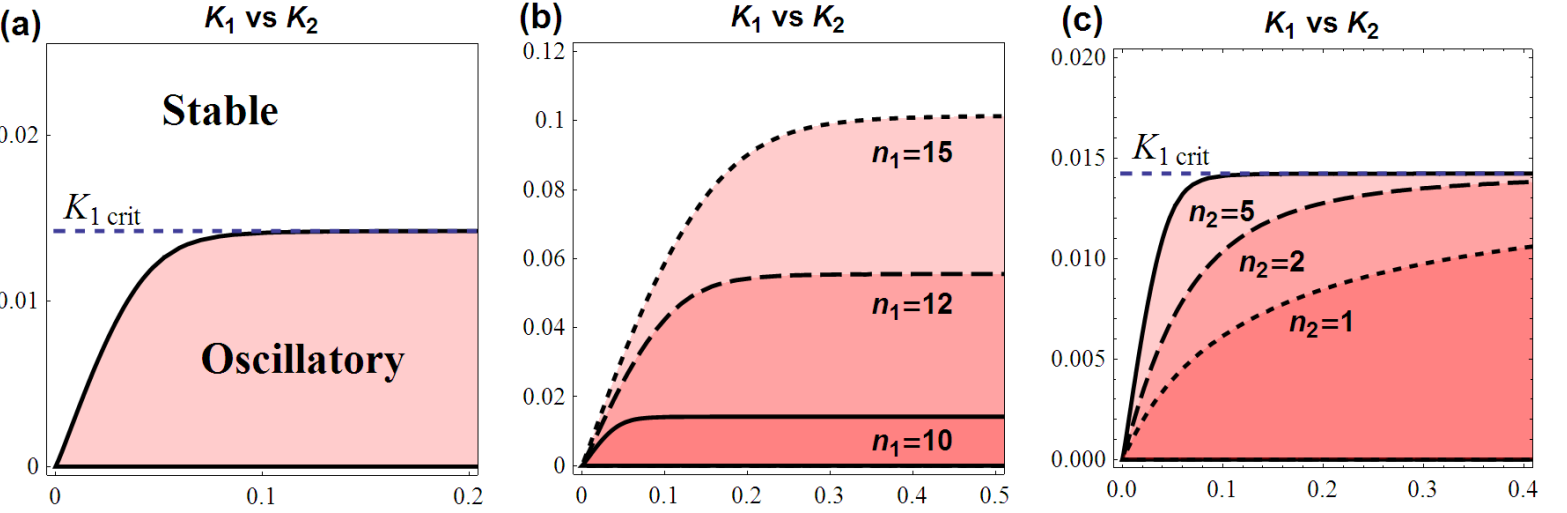
**(a) *K*_1 _vs. *K*_2 _bifurcation diagram at *n*_1 _= 10, *n*_2 _= 5 (using *k*_1 _= 1, *k*_2 _= 2, *k*_3 _= 1, *k*_d1 _= 1.2, *k*_d2 _= 3, *k*_d3 _= 2); (b) Bifurcation diagram for increasing *n*_1 _at *n*_2 _= 5; (c) Bifurcation diagram for increasing *n*_2 _at *n*_1 _= 10**.

Note that in this case, there exists at most one (unique) intersection point between *f*(*M*_2_) and *g*(*M*_2_) within *U*, indicating a simple binary separation of the *K*_1_-*K*_2 _bifurcation diagram into stable and oscillatory regions. A typical *K*_1_-*K*_2 _bifurcation profile for  is illustrated in Figure 9a. We refer to the feedback loop involving *K*_1 _and *K*_2 _as loop *L*_1 _and *L*_2_, respectively.

As loop *L*_1 _is relaxed (larger *K*_1_), sustained oscillation becomes more difficult to obtain at stronger *L*_2 _(indicated by raised minimum *K*_2 _possible for oscillation). Stability is most likely under weak *L*_1 _coupled with strong *L*_2_. Oscillations, on the other hand, are most likely at weak *L*_2 _coupled with strong *L*_1_; shifting system dynamics to being stable at strong *L*_1_, however, requires *L*_2 _to be very strong too. Dynamical behaviours are summarised in Table 1 based on combinations of the individual loop's strength.

**Table 1:** Dynamical Behaviour of Doubled-loop System

**L_1_**			
***L*_2_**	**Sufficiently Weak** (*K*_1 _> *K*_thresh_)	**Weak** (large *K*_1_)	**Strong** (small *K*_1_)
**Weak **(large *K*_2_)	**S**	S/O	**O**

**Strong **(small *K*_2_)	**S**	**S**	S/O

**Very Strong **(v. small *K*_2_)	**S**	S	S

#### K_1_'s Critical Value

Figure 9a also shows that there exists a critical value for *K*_1 _above which the system must be stable. This means stability is guaranteed if *L*_1 _is sufficiently weak, regardless of the nature of the second loop *L*_2_. Unlike *L*_1_, stable as well as oscillatory dynamics can be obtained at any strength of *L*_2_, given the proper specification of *L*_1 _(Table 1).

The critical *K*_1crit _mentioned above was found to have the exact same expression as the *K*_t1hresh _in (13)



Note that *K*_1crit _is independent of *L*_2_'s specification, whereas it increases with *n*_1_. As a result, higher *n*_1 _enhances oscillatory behaviour due to the expansion of the oscillatory region. Increase *n*_1 _therefore enables oscillatory exhibition at weaker *L*_1 _(Figure 9b). Moreover, we found that increasing *n*_2 _does also expand the oscillatory region (Figure 9c), enabling oscillatory exhibition at stronger *L*_2_. Therefore, for a coupled-loop system, raising the Hill coefficient of any loop tends to enhance oscillatory behaviour.

##### Effects of turnover parameters

**Figure 10:**
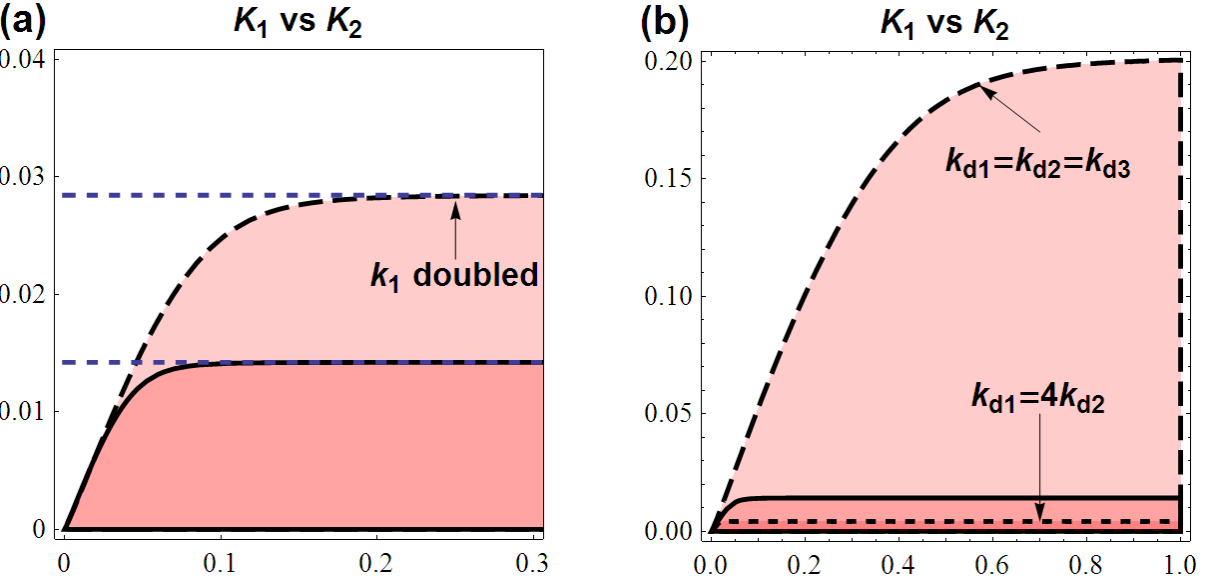
**Effects of the synthetic and degradation parameters on the two-parameter *K*_1 _vs. *K*_2 _bifurcation diagram**. **(a) **Comparison of the *K*_1 _vs. *K*_2 _bifurcation diagram between the reference parameter set (*k*_1 _= 1, *k*_2 _= 2, *k*_3 _= 1, *k*_d1 _= 1.2, *k*_d2 _= 3, *k*_d3 _= 2) and when a synthesis rate (*k*_1_) is doubled; **(b) **Comparison of the *K*_1 _vs. *K*_2 _bifurcation diagram for 3 parameter sets: the reference set above (solid); when the degradation rates are all equal = 1.2 (dashed); and when one degradation rate is much greater than another (dot).

The above equation indicates that *K*_1crit _increases proportionally with the synthesis rates. This causes the oscillation region to approximately increase by the corresponding proportion. Oscillatory dynamics is now achievable at higher *K*_1 _given fixed *K*_2 _(Figure 10a). On the other hand, comparable degradation rates (*k*_d1 _≈ *k*_d2 _≈ *k*_d3_) leads to low *B *(see section 1.1.6 in Additional file 1) and as a result raises *K*_1crit_. Particularly, *K*_1crit _is maximised when this comparable rate is minimised. Whereas, if these parameters are different by many folds, *K*_1crit _is small and so is the corresponding oscillatory region. Figure 10b compares three scenarios in this case. The parameter set with *k*_d1 _= *k*_d2 _= *k*_d3 _= 1.2 obtains the largest oscillatory region while setting *k*_d1 _= 4*k*_d2 _has it significantly diminished.

#### Three-species, Doubled-loop System 

An alternative doubled-loop system is considered here in which the first and the last pathway steps are inhibited (see Figure 3e for the schematic diagram and model equations). Follow the analytical methodology in section 2; we obtain the following stability condition for the system

(21)

with

 and , where coefficients *a*_0_, *a*_1_, *a*_2 _and *b*_1 _are given in the supplementary material (section 4.3, Additional file 1).

#### K_1_-K_3 _Bifurcation Diagram

Similar analysis was carried out on the two-dimensional *M*_1_-*M*_3 _coordinate to determine the ranges of *M*_3 _satisfying condition (21). Figure 4b shows that, in this case, *g*(*M*_3_) is a concaved-up parabola instead of a straight line like *g*(*M*_2_) in the previously considered system . Nevertheless, there exists still at most one intersection point between *f*(*M*_3_) and *g*(*M*_3_), resulting in a similar bifurcation pattern for the *K*_1 _vs. *K*_3 _diagram as in  (Figure 4a). Moreover, the critical value for *K*_1 _discussed previously is found to have the same form here. This again confirms that incorporation of additional feedback loop does not affect *K*_1crit_, regardless of the location of the added loop. Moreover, the first loop's Hill coefficient (*n*_1_) also has its RGS unchanged: RGS = (0, *B*]. The two-parameter *K*_1 _vs. *K*_3 _bifurcation diagrams were constructed based on the following relation:



Here, we compare the bifurcation profiles between two doubled-loop systems  and . To facilitate this, we impose *K*_2 _= *K*_3 _and *n*_2 _= *n*_3_. The line *g*(*M*_2_) is superimposed on the *M*_1_-*M*_3 _plane (by setting *M*_2 _≡ *M*_3_) and indicated by the thin line in Figure 4b. Note that that *g*(*M*_2_) and *g*(*M*_3_) meets on the *M*_1_-axis. Moreover, the slope at this point for *g*(*M*_3_) is always steeper than *g*(*M*_2_), suggesting a higher lower bound for *M*_3 _for stability. The implication is: given the same set of parameter values, adding loop *L*_3 _results in a larger stability region (a smaller oscillatory region) than adding loop *L*_2_, therefore better enhance system stability. On the other hand,  is more likely to exhibit oscillatory dynamics than .

#### Three-species, Multiple-loop System 

In this section, we consider the system structure which incorporates all three feedback loops imposing on all pathway steps (see Figure 3g for the circuit diagram and model equations). The analysis becomes more complicated, as a result. For this multiple-loop system, the stability condition is given by



**Figure 11:**
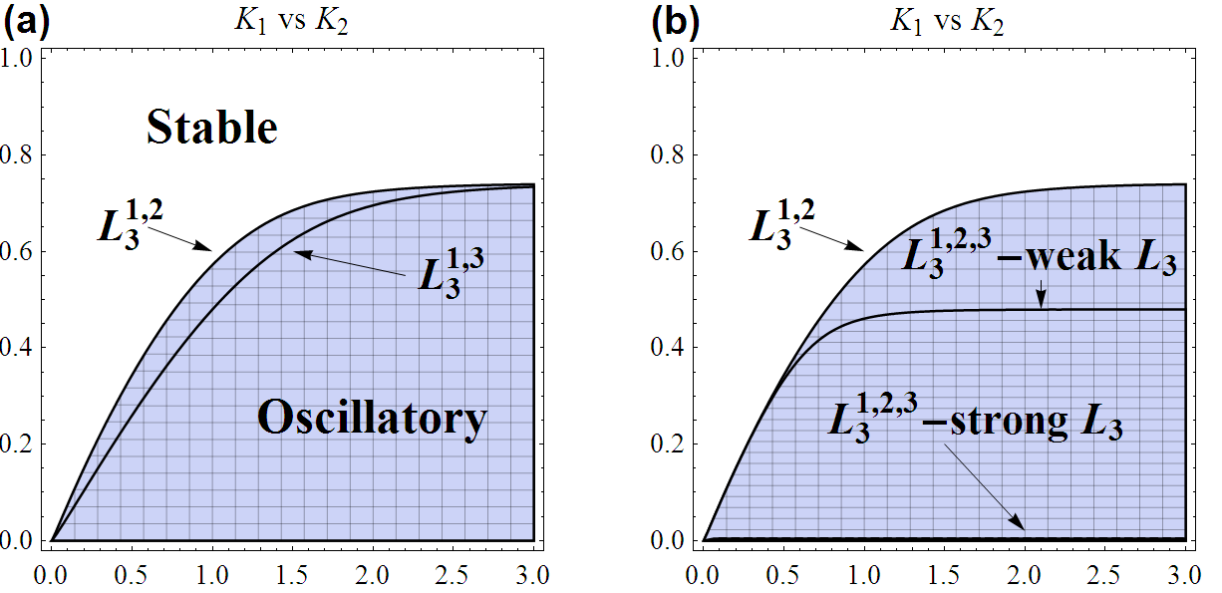
**Comparison of bifurcation diagram between coupled-loop systems**. **(a) **Bifurcation diagram comparison between  and  systems (we used *n*_1 _= 13, *n*_2 _= *n*_3 _= 5 and the reference parameter set). **(b) **Comparison of bifurcation diagram between systems with only first two loops, with all three loops but weak *L*_3_, and strong *L*_3_.

Due to space restriction, we give the explicit forms of function *f *and *g *in the supplementary material (section 4.4, Additional file 1). *K*_1 _was derived as a function of the remaining model parameters:



Following the similar methodology laid out above, we were able to compute bifurcation diagrams for any pair of feedback strengths (*K*_1_vs. *K*_2_, *K*_1 _vs. *K*_3_, and *K*_2 _vs. *K*_3_). In all the cases, it is found that having extra third loop always increases the stability of the system, illustrated by expansion of the stability region on bifurcation diagrams. The likelihood of obtaining oscillatory dynamics is directly controlled by strengths of the loops in effect, with stronger feedback loops always result in smaller oscillatory region. Figure 11b compares the bifurcation profiles of the doubled-loop and three-loop systems.

#### Four-species, Coupled-loop System

To further understand the effects of loops coupling in negative feedback systems, we examine a system consisting of four species with two loops in action, denoted  (see Figure 3l for the circuit diagram and model equations). Our derivation arrives at the stability condition for this system, given below

(22)

In this case *f*(*M*_2_) still have the usual form

(23)

with *A *as in equation (15). However, we have

(24)

where the coefficients *a*_0_, *a*_1 _and *a*_2 _are given in the supplementary material (section 4.5, Additional file 1).

In a similar fashion, to construct bifurcation diagram of the loops' feedback strengths, we need to determine the ranges of *M*_2 _that satisfies (22) via examining the curves in (23) and (24) on the *M*_1_-*M*_2 _coordinate. System stability occurs over the ranges of *M*_2 _such that *f*(*M*_2_) lies below *g*(*M*_2_), while oscillatory dynamics reigns over the remaining ranges.

As expected, system extension greatly complicates the analysis due to the increased number of parameters and the increased complexity of *g*(*M*_2_).

##### Loops Coupling lowers Hill coefficients for Oscillations

To find the RGS for the Hill coefficients *n*_1 _and *n*_2_, we determine on the two-parameter Hill coefficients *n*_1_-*n*_2 _plane regions that give rise to system stability regardless of the feedback loops' strengths and other parameters. These regions can be referred to as the Regions of Guaranteed Stability for *n*_1 _and *n*_2_.

**Figure 12:**
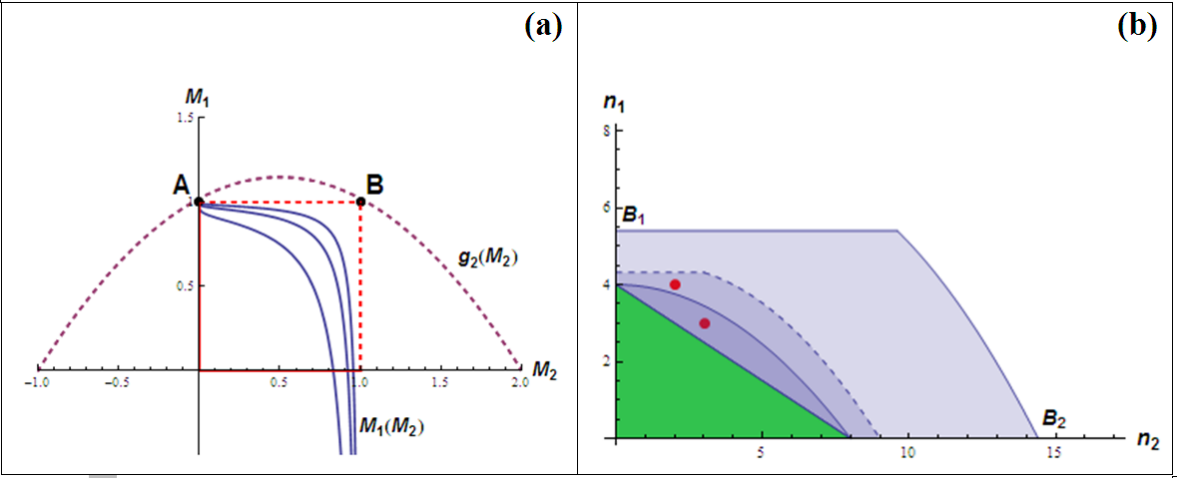
**(a) The *f*(*M*_2_) and *g*(*M*_2_) on the *M*_1_-*M*_2 _plane**. Several *f*(*M*_2_) with 3 different *K*_2 _values. The critical *g*(*M*_2_) curve is indicated by the dashed parabola which meets the unit square U at corner points A and B. **(b)** The RGS regions shown on the *n*_1_-*n*_2 _plane.

From Figure 12a we can see that condition (22) will always be satisfied if *g*(*M*_2_) always lies above the unit square U and therefore above *f*(*M*_2_) for all *K*_2_. The RGS for *n*_1_, *n*_2 _can thus be found by solving the following system of inequalities,

(25)

Put , which only depends on the degradation parameters. We obtain the RGS as containing those points (*n*_2_, *n*_1_) on the *n*_1_-*n*_2 _plane such that

(26)

where *B*_1 _and *B*_2 _have the same form as in (16) and (17), respectively. Specifically,



This region is presented in Figure 12b as the marked area bounded by the axes (with *B*_1 _and *B*_2_). Figure 12b shows the RGS region for three different sets of the degradation rates. Interestingly, we found that for arbitrary values of degradation parameters, the corresponding RGS always contain the 8 by 4 triangular, indicating that any (*n*_1_, *n*_2_) inside this triangular guarantees system stability regardless of all other model parameters including the feedback strengths. On the other hand, for (*n*_1_, *n*_2_) outside the triangular, we can always find a set of *k*_*di *_(*i *= 1,..,4) so that its RGS does not contain (*n*_1_, *n*_2_), giving rise to unstable system equilibrium.

Recall that in the cases of single-loop systems considered before, there exist lower bound conditions for the Hill coefficients if oscillatory dynamics is to be obtained. For example, *n*_1 _must > 4 for , and *n*_2 _must > 8 for . Feedback loops coupling, however, effectively removes these constraints for the Hill coefficients. In fact, sustained oscillation is now achievable for any value of *n*_1 _(*n*_2_) given proper choice of *n*_2 _(*n*_1_). As a result, sustained oscillation can occur at much more biologically plausible values of *n*_1_, *n*_2_; e.g. (*n*_1_, *n*_2_) = (3, 3) or (2, 4), indicated by the dots in Figure 12b.

It is important to note that the RGS for (*n*_1_, *n*_2_) solely depends on only the degradation rates. Variation on these rates affects its size and location. *B*_2 _is maximised if among the degradation parameters, one is many folds greater than another (*k*_di _>> *k*_d*j *_with *i*, *j *∈ {2,3,4}). Similarly, *B*_1 _is maximised if *k*_di _>> *k*_d*j*_. On the other hand, *B*_1_, *B*_2 _are lowest when *k*_*di *_>> *k*_*dj *_with *k*_d1 _≈ *k*_d2 _≈ *k*_d3 _≈ *k*_d4_. Therefore, reducing any degradation rate to extreme low or high level will expand the RGS, resulting in system stability for wider range of Hill coefficients. Oscillation, consequently, is enhanced when the degradation rates are kept comparable between the system species.

#### Loops Coupling Generates 13 different K_1_-K_2 _Bifurcation Patterns

**Figure 13:**
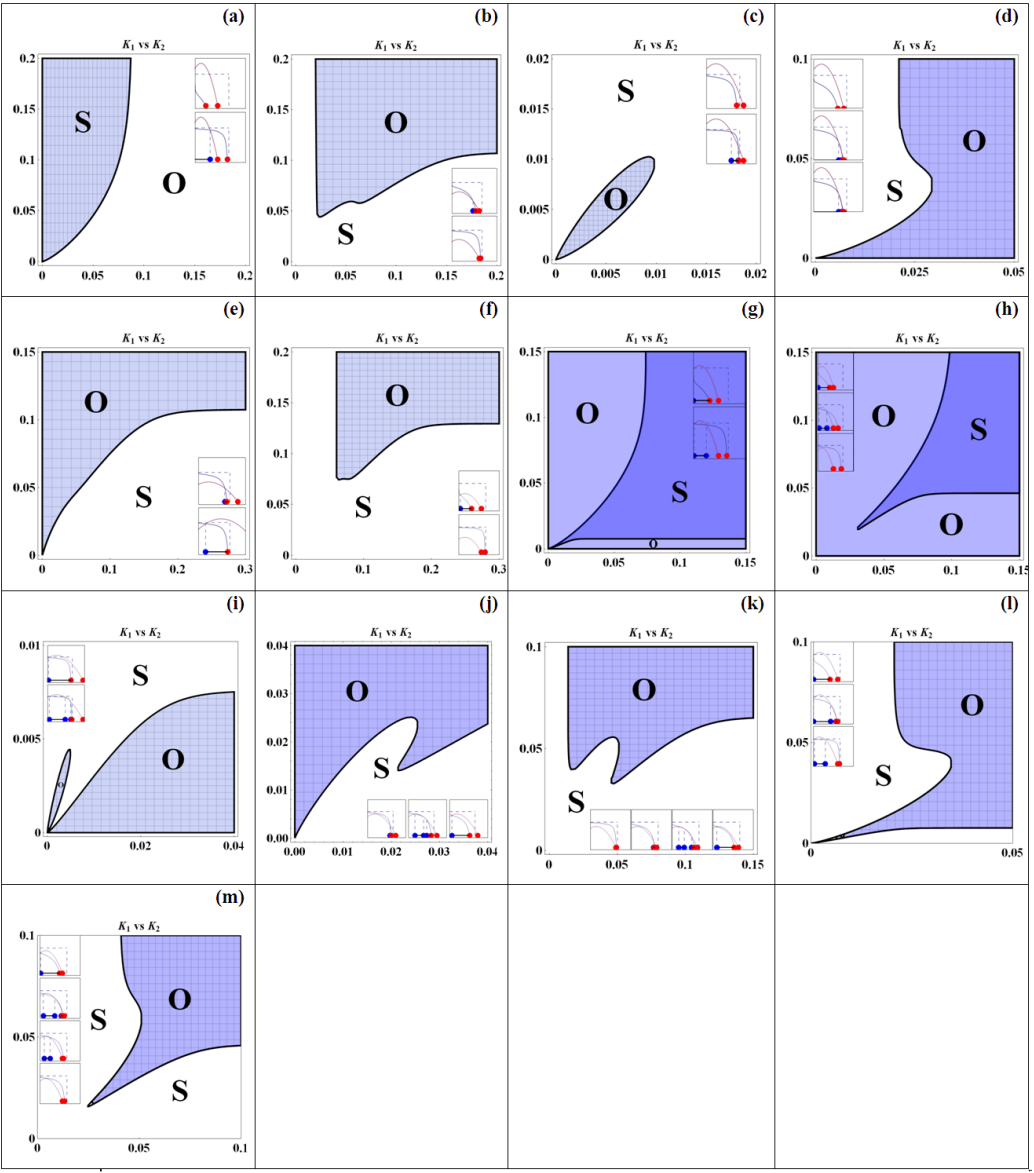
**13 different patterns of the two-parameter *K*_1 _vs. *K*_2 _bifurcation diagrams**. S denotes stable regions and O denotes oscillatory regions. For each bifurcation diagram, the inset graphs indicate the different scenarios (number of intersection points) of the functions *f*(M_2_) and *g*(M_2_).

If (*n*_1_, *n*_2_) lies outside the RGS region, *g*(*M*_2_) must cross U and therefore must intersect with *f*(*M*_2_) at least once at some value of the feedback strength indicator *K*_2_. Unlike the previously considered systems where only one intersection point is detected, the number of intersection points in this case could be up to three. This provides a rich variety of different bifurcation profiles for the system. In fact, we identify a total of 13 distinct patterns of bifurcation on the *K*_1 _vs. *K*_2 _bifurcation plane; each pattern for one choice of the Hill coefficients. These 13 patterns are displayed in Figure 13.

These bifurcation diagrams differ in their characteristics: the shapes of the stable and oscillatory regions. For example, Figure 13a shows a simple bifurcation profile: for each and every value of *K*_1_, the system displays oscillations over a range of *K*_2 _with a lower bound but no upper bound. Figure 13c shows a similar feature but the range of *K*_2 _for oscillations is now bounded by both the lower and upper bounds; moreover, this range only exists for a certain range of *K*_1_. Figure 13k displays even more intricate bifurcation characteristics: as the parameter *K*_1 _moves up the vertical axis, the corresponding set of values of *K*_2 _for oscillations continually changes with no, one bounded range, two bounded ranges, and one unbounded range. This indicates the complexity between the feedback loops' strengths in contributing to shaping up the dynamics of the system, as a whole.

### End-product Utilisation

In the preceding model systems, reduction of model species was assumed to occur only through degradative processes (decay and modification). However, species' reduction could also occur via other mechanisms. We consider here the scenario where the end-product (inhibitor), besides being degraded, is due to be consumed by the cell for synthesising other cellular components. Notable examples are of common amino acid biosynthesis pathways in which the amino acid (pathway's end-product) is utilised by the cell for protein synthesis [33-35]. Earlier work [16,35] suggests that the change of the end-product in this manner has important effects on the dynamical stability of the system. However, these work were numerical; there are no analytical analyses of this effect.

Although the inclusion of *g *complicates our analysis, we were able to obtain the stability condition for the system in simple form similar to (11) (see section 3 in Additional file 1 for detailed derivation)



with *B*_g _is given by (note that *A *and *B *are as in (15) and (16) of system ):



As shown in Additional file 1, section 3, for the system to have equilibrium, the utilisation rate *g *must not exceed a critical value

(27)

#### End-product utilisation enables oscillations at any Hill coefficient

**Figure 14:**
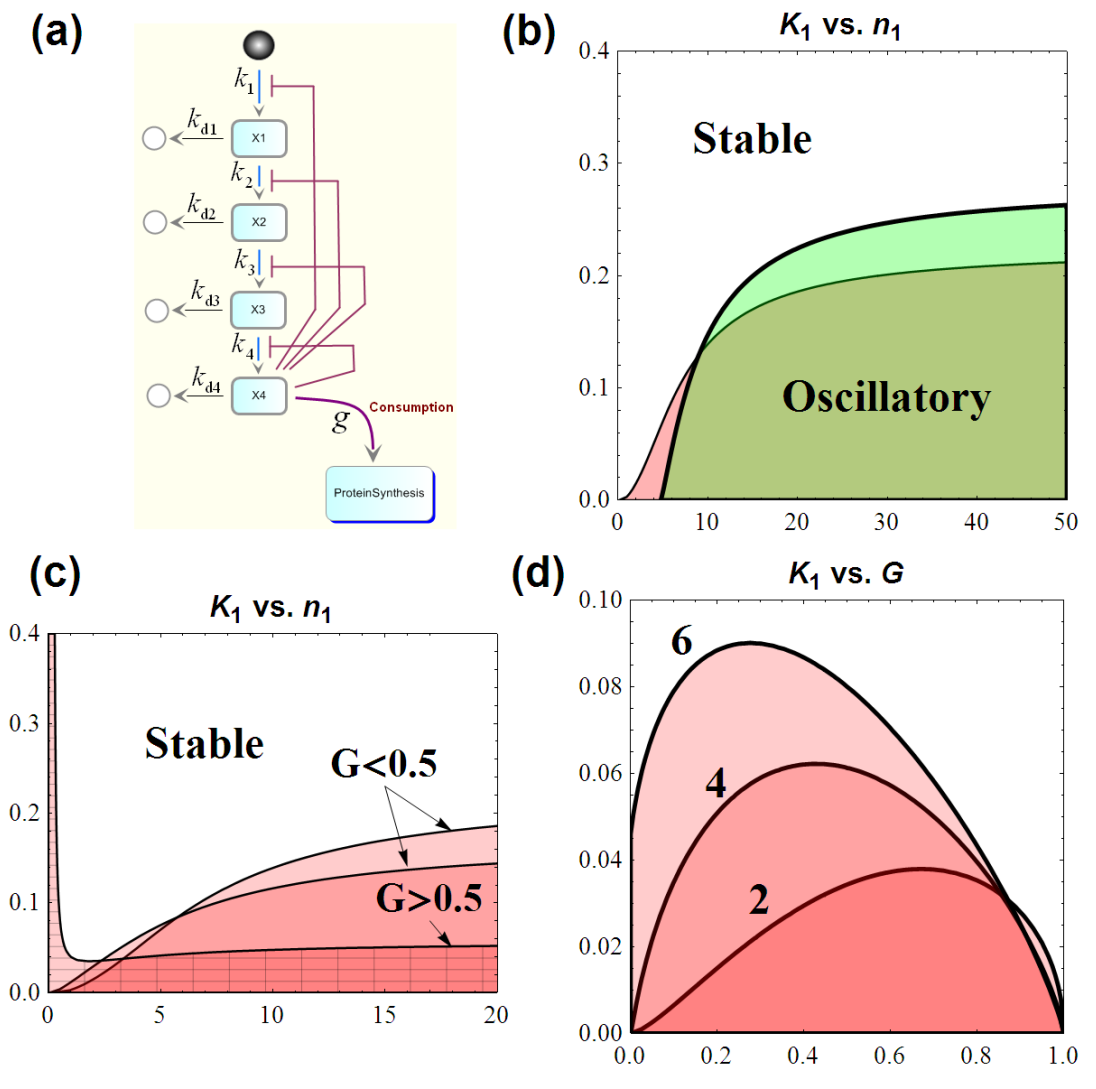
**(a) Schematic diagram of the negative feedback system with end-product utilisation**. (b, c, d) The *K*_1 _vs. *n*_1 _bifurcation diagram for the system with end-product utilisation.** (b) **Bifurcation diagram comparison between system without end-product utilisation and one with utilisation on *K*_1_-*n*_1 _plane (*G *= 0.2 was used). **(b) **Bifurcation diagram of system with utilisation for increasing *G *(*G *= 0.2, 0.4, 0.8 were used). **(c) **Two-parameter *K*_1 _vs. *g *bifurcation diagrams for *n*_1 _= 2, 4 and 6; oscillatory regions are in red (parameter set *k*_1 _= 1, *k*_2 _= 2, *k*_3 _= 1, *k*_4 _= 1, *k*_d1 _= 1.2, *k*_d2 _= 3, *k*_d3 _= 2, *k*_d4 _= 1 was used).

With (27), it is easy to check that *B*_g _< 1 for all *n*_1 _and *g*. Hence, for any *n*_1_, stability condition (3.24) can be breached by choosing *K*_1 _sufficiently large (see Additional file 1, section 1.1.3), and so the system is destabilised (oscillatory). Moreover, this is true for arbitrary value of *g *> 0. The interesting implication here is that, unlike system  where sustained oscillation is only attainable for certain *n*_1 _(*n*_1 _must be greater than 4), the inclusion of *g*, even small, has enabled the system to attain oscillation at any *n*_1_. End-product utilisation therefore allows oscillatory dynamics at low cooperativity level. This is demonstrated in Figure 14b where bifurcation diagrams on the *K*_1_-*n*_1 _plane are compared for system with (thick line) and without (thin line) end-product utilisation. The bifurcation diagrams were constructed based on the threshold *K*_1 _which we calculated to be (see Additional file 1, section 3 for the derivation):

(28)

This threshold approaches a critical value as *n*_1 _increases; *K*_1crit _is given by (1-*G*)/*A *which is smaller than *K*_1crit _of  (1/*A*). This indicates in order to achieve oscillation; the feedback strength of the system with end-product utilisation must generally be stronger (Figure 14b).

#### Effects of g on systems dynamics

We expect that change in *g *would bring change in the dynamical characteristics of the system. We found that this change comes about in an interesting way. As *g *(*G*) increases, *K*_1crit _reduces, causing shrinking of oscillatory region, especially at high *n*_1 _(Figure 14c). Therefore, higher utilisation of the end-product generally requires stronger feedback loop if oscillation is to be obtained. However, when *G *exceeds 0.5, the oscillatory region changes its shape significantly resembling an *L *shape, indicated by the crossed area in Figure 14c (Figure 14c compares when *G *= 0.8 against *G *< 0.5). Now at low *n*_1_, oscillatory region is greatly expanded, enabling oscillation at a much wider range of *K*_1_.

We also present on Figure 14d the two-parameter *K*_1 _vs. *G *bifurcation diagrams for *n*_1 _= 2, 4 and 6. In this case, higher cooperativity generally gives rise to larger oscillatory region, consequently promoting oscillation. We observe an intermediate value of *G *(and so *g*) for which oscillation is most likely (widest range of *K*_1_) while at low and high *G *the system tends to be more stable. This is consistent for all three plotted *n*_1_. As *n*_1 _increases, this intermediate *g *moves further left in its spectrum (between 0 and *g*_c_)

To sum up, we showed that end-product utilisation enhances sustained oscillation at low cooperativity level while it enhances stability at high cooperativity. However, it is important to note that raising the utilisation level does not always further these enhancements. In fact, there exists an intermediate rate for utilisation at which sustained oscillation is detected most likely, while less likely at other rates.

### Biological Examples

Restricted by the paper's scope, we only present here two biological examples from the literature where useful insights can be readily obtained by applying the analysis outlined in this paper. These are (1) the Hes1 oscillator which plays an important role during somitogenesis of vertebrates and (2) the Tryptophan operon system responsible for regulatory production of tryptophan in *Escherichia coli*. The former system exemplifies single-loop system while the later is an instance of multiple-loop system.

#### The Hes1 Oscillator

**Figure 15:**
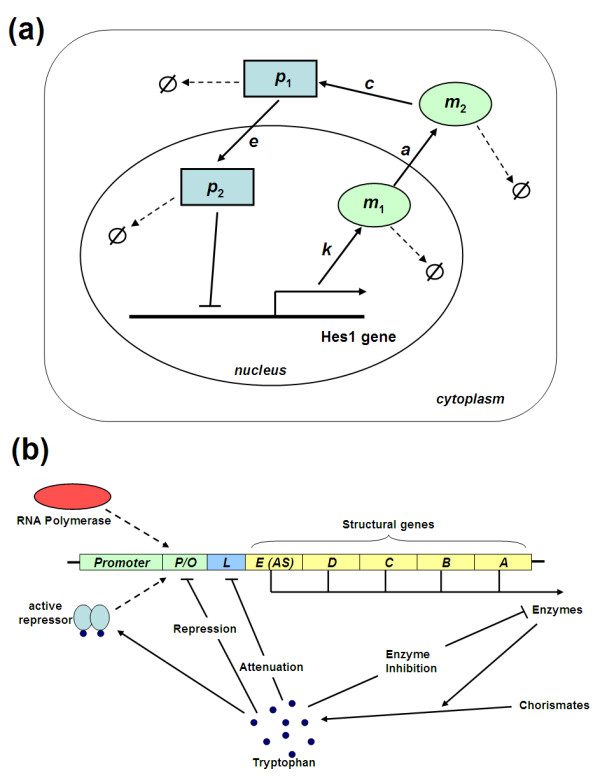
**(a) Schematic representation of the Hes1 network**. **(b) Schematic diagram of the tryptophan operon system**. 5 genes are denoted as E (AS), D, C, B and A. P, O, L denotes the promoter, operator and leader region, respectively. Blunt arrow represents inhibition while normal arrow represents activation.

A wide range of cellular phenomena have their activities centred on oscillations [36,37]. One such notable example is vertebrate somitogenesis. This is a developmental process in which the vertebrate embryo becomes segmented by the regular sequential assignment of mesodermal cells to discrete blocks [38]. Experimental evidence reveals the basic helix-loop-helix (bHLH) transcription factor Hes1 as an important cyclic gene driving this oscillations [6,39,40]. These studies showed that the oscillatory expression of the bHLH factor Hes1 is regulated by a direct negative feedback loop whereby Hes1 represses the transcription of its own coding gene (Figure 15a)

A few models have been developed for this network [6,41-44]. Here we base our analysis on a model suggested by Zeiser et al. [43] which consists of four ordinary differential equations involving Hes1 mRNA and protein and incorporates their transportation processes between the nucleus and cytoplasm. Using notations in the original paper, the model equations are given below,

(29a)

(29b)

(29c)

(29d)

Here *m*_1 _and *m*_2 _represents the concentration of Hes1 mRNA before and after being transported from the nucleus to the cytoplasm, respectively; while *p*_1 _and *p*_2 _are the concentration of Hes1 protein before and after being transported from the cytoplasm to the nucleus, respectively. Equation (29a) describes the synthesis of mRNA in the nucleus. The mRNA is then transported into the cytoplasm, described by (29b). Translation into protein is specified by (29c) while (29d) represents transportation of the protein into the nucleus where it represses its own transcription. Parameters *b *and *a *denote the decay and modification rates for mRNA respectively; while *d *and *e *are used for the protein. To simplify the analysis, Zeiser et al. assumed the decay rates (*b *and *d*) as being identical for both forms of the mRNA and protein. By fixing *b *= 0.028, *d *= 0.031 and under condition of having oscillation period about 120 min, all determined in Hirata et al. [6], Zeiser et al. estimated a set of fitting parameters, displayed in Table 2.

**Table 2:** Parameter values for the Hes1 oscillator estimated in Zeiser et al. [43]

*b*	*d*	*A*	*e*	*c*	*H*	*k*	*h*
**0.028**	**0.031**	0.05	0.09	0.2	10	30	6.2

**Figure 16:**
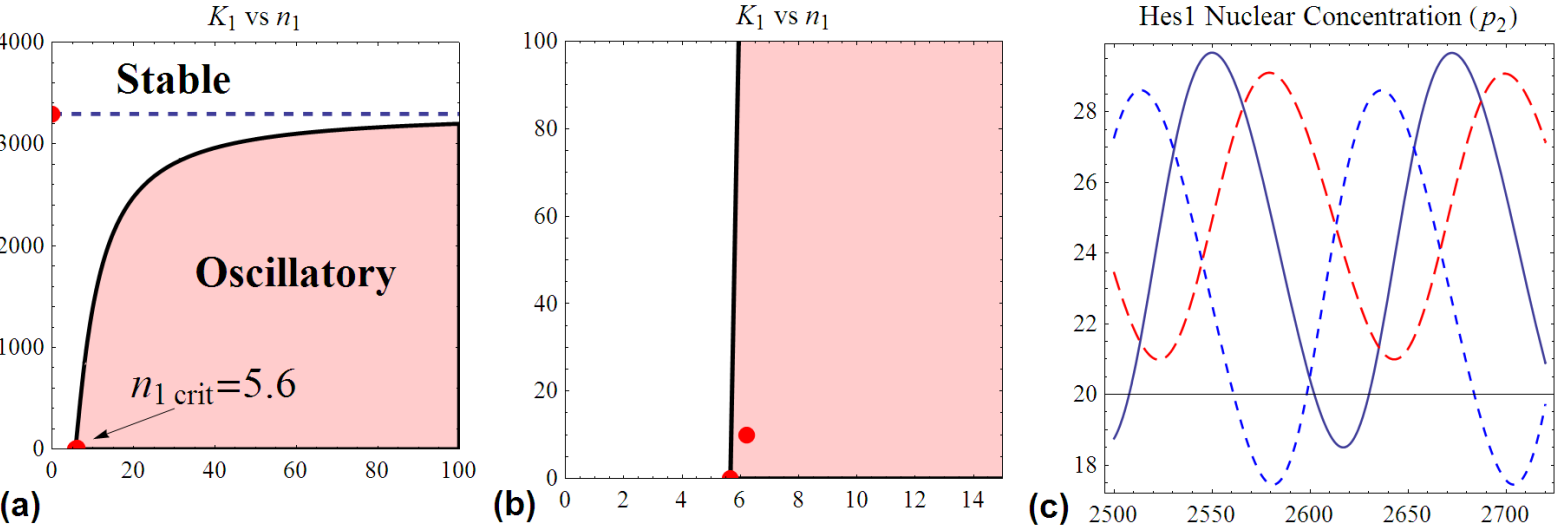
**The Hes1 oscillator**. **(a) **Two-parameter *K*_1_-*n*_1 _bifurcation diagram for the Hes1 oscillator. **(b) **Close-in of the bifurcation diagram with Zeiser et al.'s point highlighted in red. **(c) **Temporal revolution of Hes1 nuclear concentration (*p*_2_) for three feedback strengths: *K*_1 _= 10 (black, solid); *K*_1 _= 0.1 (blue, dot) and *K*_1 _= 100 (red, dash).

It is easy to see that model (29) is just a special case of system model  analysed earlier with parameters adapted and presented in Table 3. Consequently, the threshold Hill coefficient is computed based on equation (16) using only the degradation parameters gives a value of about 5.6. This means for the Hes1 oscillator to be oscillating at all, the Hill coefficient must be greater than 5.6. The Hill coefficient used by Zeiser et al., 6.2 is quite close to this minimum value (Table 3 and Figure 16b). We constructed the two-parameter *K*_1_-*n*_1 _bifurcation diagram in Figure 16a. The threshold feedback strength can also be readily calculated from equation (15), as *K*_1thesh _= 1/*A *= 3296. Thus, for the Hes1 system to be an oscillator, the necessary condition for *K*_1 _is that *K*_1 _< 3296, given other parameters' values in Table 3. Moreover based on Figure 16a, at *n*_1 _= 6.2, viable range of feedback strength for the Hes1 oscillator is 0 <*K*_1 _< 207.6. Zeiser et al. therefore used a quite small *K*_1 _(i.e. 10). Interestingly, we find that variation of the feedback strength (*K*_1_) has little effect on the oscillation period. Figure 16c compares the temporal change of Hes1 protein concentration, *p*_2 _in (29), for the parameter set in Table 2 and when feedback strength is 100-fold stronger (*K*_1 _= 0.1), and 10-fold weaker (*K*_1 _= 100). So in fact, constraining the oscillation period to be 120 mins can gives rise to many more suitable parameter sets other than one in Table 3.

**Table 3:** Values for the parameters of the general system  adapted from Table 2.

*k*_1_ (*k*)	*k*_2_ (*a*)	*k*_3_ (*c*)	*k*_4_ (*e*)	*k*_d1_ (*b*+*a*)	*k*_d2_ (*b*)	*k*_d3_ (*d*+*e*)	*k*_d4_ (*d*)	*K*_1_ (*H*)	*n*_1_ (*h*)
30	0.05	0.2	0.09	0.078	**0.028**	0.121	**0.031**	10	6.2

#### The Tryptophan Operon System

The tryptophan operon system in *E. coli *controls the production of tryptophan amino acid inside the cell. Key molecular processes include transcription, translation and synthesis of tryptophan. To regulate these processes, the tryptophan operon utilises three negative feedback mechanisms: transcriptional repression, attenuation, and enzyme inhibition [8,45].

The transcription process is initiated as RNA polymerase binds to the promoter. However, when the activated form of repressor which is induced by the attachment of two tryptophan molecules become abundant, it will bind to the operator site and block RNA polymerase from binding to the promoter, thereby, repressing transcription and forming the first feedback loop. Furthermore, transcription can also be attenuated depending on the level of intracellular tryptophan and is controlled by the leader region sitting between the operator and the genes (Figure 15b). This attenuation makes up the second feedback loop. The tryptophan operon consists of five structural genes positioned consecutively after the leader region. These genes code for five polypeptides that make up enzyme molecules in the form of tetramers, which in turn catalyse the synthesis of tryptophan from chorismates [8,29,45,46].

Anthranilate synthase (AS) is the enzyme catalysing the first reaction step in the tryptophan synthesis pathway. The pathway end product tryptophan is fedback to inhibit anthranilate synthase activity if tryptophan level is high. Enzyme inhibition therefore forms the third negative feedback loop in the tryptophan operon system.

(30a)

(30b)

(30c)

We set up a simple three-species model for the tryptophan system as in equations (30). The state variables are the mRNA (*M*), the AS enzyme (*E*) and the tryptophan amino acid (*T*). Each negative feedback loop is modelled using a Hill function; *O*_t _is the static total operon concentration; *k*_1_, *k*_2_, *k*_3 _represent transcription rate, translation rate and tryptophan synthesis rate, respectively; *k*_d1_, *k*_d2_, *k*_d3 _are the degradation rates (aggregated parameters combining the decay rate and dilution rate due to cell growth). Consumption of tryptophan for protein synthesis is simply assumed to follow first order kinetics and represented by the last term of equation (30c). Parameter values are adapted from [47,48] and tabulated in Table 4.

**Table 4:** Parameter values for the Tryptophan operon system.

**Parameters**	**Value**
*k*_1_	50 min^-1^
*k*_2_	90 min^-1^
*k*_3_	60 min^-1^
*k*_d1_	15.01 min^-1^
*k*_d2_	0.01 min^-1^
*k*_d3_	0.01 min^-1^
*K*_1_	3.53 μM
*K*_2_	0.04 μM
*K*_3_	810 μM
*n*_1_	1.92
*n*_2_	1.72
*n*_3_	1.2
*G*	25
*O*_*t*_	0.00332 μM

It can be seen that model (30) is a case of the general multiple-looped  considered in the section "Coupled-loop systems". We applied the analysis to the tryptophan operon system for the parameter set in Table 4. Furthermore, for each parameter *p *in Table 4, we varied it over a wide range around its nominal value (from *p *× 10^-2 ^to *p *× 10^2^) while fixing the other parameters. Under all these scenarios, system stability was always obtained. Given the nominal values of the synthetic and degradation rates, the system failed to demonstrate oscillations even at very high Hill coefficient values (> 50) and at weak or strong feedback loops. This suggests that the tryptophan system is extremely stable.

The highly stable property of the tryptophan system is probably underlined by the fact that it is regulated by multiple feedback loops in concert. In addition, the system's degradation rates *k*_d1_, *k*_d2 _and (*k*_d3_+g) are significantly different to each other as shown in Table 4. This disparity in value of the degradation rates, as discussed earlier, greatly enhances system stability. On the other hand, by adjusting *k*_d1_, *k*_d2_, *k*_d3 _and g so that *k*_d1 _≈ *k*_d2 _≈ *k*_d3_+g, oscillatory behaviour can now be observed at much lower Hill coefficient and at appropriate feedback strength of the loops. For example, setting *k*_d1_= *k*_d2_= *k*_d3_+g = 15 can give rise to oscillatory dynamics with *n*_2_, *n*_3 _as in Table 4 and *n*_1 _as low as 8.5.

## Summary and conclusion

Previous studies [14-19] have looked mainly at the effect of cooperativity level on system dynamics, while largely neglecting the effects of feedback strength. Furthermore, most of these analyses were carried out numerically; those with analytical approaches were however often done under great simplification for model equations such as stringently assuming that all degradation parameters are identical [15,19]. The major contributions from our study are summarised and discussed below.

### Threshold feedback strength

For single-loop systems where inhibition is fedback by the end-product on the first reaction step, i.e. the original Goodwin system, it was found that oscillatory behaviour is only obtainable if the feedback loop is sufficiently strong. Otherwise, the system is stable and achieves steady state. Switching between these dynamics occurs through a Hopf bifurcation. We derived an explicit, analytical form for the feedback strength's bifurcation point which can be straightforwardly computed if the other model parameters are known. Interestingly, this threshold strength was found to follow a saturation trend and approaches a critical level as the Hill coefficient increases. We further showed that this critical feedback strength equals the ratio of the product of the degradation rates and the product of the synthesis rates. So for a system with feedback strength weaker than this critical level, system stability is guaranteed regardless of how high the Hill coefficient is.

Studying the two-parameter bifurcation diagram with the feedback strength and the Hill coefficient as parameters revealed that as the Hill coefficient is raised, sustained oscillation can be obtained over a wider range of feedback strength; suggesting that higher cooperativity level tends to enhance the probability of demonstrating sustained oscillations. This result is in line with previous results from Tyson & Othmer [15] and Goldbeter [49].

### Threshold Hill coefficient

Assuming identical degradation rates, previous studies of the Goodwin system have identified a threshold value for the Hill coefficient lower than which, sustained oscillation is unachievable. Under this over-simplifying assumption, the threshold cooperativity level was found only dependent of the length of the system pathway. However, such a similar threshold Hill coefficient has not been analytically determined for systems with general, arbitrary degradation rates. We explicitly derived this threshold for the three and four-species systems, which turns out to be rather simple, symmetrical functions of only the degradation rates and are not influenced by the synthesis rates. More importantly, the threshold reaches its minimum when all the degradation rates are equal. The threshold Hill coefficient obtained under identical degradation rates, therefore, provides a lower bound for that of the general case. We further showed that for the systems with Hill coefficient exceeding the threshold value, it can always oscillate with a properly chosen set of parameters' values. In fact, the number of parameter sets giving rise to sustained oscillations is indefinite. For the systems with more species, the threshold Hill coefficient is also explicitly derivable; however, the form of the resulting function becomes significantly more complex. Nevertheless, the above results also apply for longer systems which we confirmed using numerical simulations.

### Effects of parameters variation

Parameter sensitivity analysis revealed interesting effects of parameters (the synthetic and degradation rates) variation on the dynamical characteristics of the system. Because of the symmetry in the expressions involving the threshold feedback strength and Hill coefficient, the individual model species equally characterise the system bifurcation profiles despite the fact that the feedback loop is only acting on the first reaction step. Specifically, increasing the synthesis rate of any model species by the same proportion results in a proportionally larger oscillatory region and hence in a system which is more likely to oscillate. In contrast to this simple linear relationship between the synthesis rates and the bifurcation profiles, the degradation rates affect system dynamics in a more intricate manner. We found that system stability is most likely when the model species are rapidly degraded while slow degradation only leads to stability if the feedback strength is significantly weak. We further showed that having comparable degradation rates between the model species promotes oscillations, whereas stability is promoted if one rate is significantly larger than another. This suggests a way to enhance system stability by unbalancing the degradation rates; preferably, towards high levels. These results are particularly helpful for the engineering of synthetic circuits with desirable dynamical behaviour, as well as for parameter estimation and optimisation.

### Feedback reallocation

For single-loop systems, reallocation of the feedback loop to inhibit a reaction step further downstream may or may not make the system more stable. Interestingly, the specific effect is determined only by the degradation rates of the model species downstream of the newly inhibited species. The dynamical properties of the new system closely resemble those of the Goodwin system with reduced length, which equals to the number of species downstream of the inhibited species. Therefore, as the loop moves closer towards the end of the pathway, the minimum Hill coefficient for oscillation is reduced. In addition, we found that feedback reallocation does not influence the critical feedback strength discussed above. This means that for a system possessing a loop weaker than this strength, its stability is ensured regardless of the loop's position and the cooperativity level.

### System extension

It has been known that lengthening the system by increasing the number of reaction steps, i.e, increasing number of model species, reduces the cooperativity necessarily required for sustained oscillations [15,19]. The implication here was that system extension enhances oscillations. However, this result was demonstrated under the assumption of identical degradation rates. When this assumption is relaxed, we found that the extended system is not always more stable. More importantly, whether it is more stable or not is attributed to the kinetics of the added species: more stable only when the added species degrades slower than it is being produced; and more oscillation-prone otherwise.

### Non-oscillatory systems

It has been known that the systems with two species are incapable of exhibiting sustained oscillations, regardless of the feedback strength and the Hill coefficient value [14,15]. Our analysis further showed that those systems with arbitrary lengths which possess a single loop inhibiting either the last step or second-last step of the pathways is also incapable of obtaining oscillatory dynamics. In addition, multiple-looped systems which include at least one loop inhibiting either the last or second last step of the pathway is also incapable of demonstrating oscillatory behaviours.

### Effects of end-product utilisation

We also investigated the situation when the pathway's end-product is used up by the cells, which is common in many metabolic pathways. Most interestingly, we showed analytically that end-product utilisation enables oscillatory dynamics **at any Hill coefficient value**. More specifically, end-product utilisation enhances sustained oscillation at low cooperativity level but enhances stability at high cooperativity level. It is important to note that raising the utilisation level does not always further these enhancements. In fact, there exists an intermediate rate for utilisation at which sustained oscillation is most likely to be detected, while being less likely at other utilisation rates.

### Effects of loops coupling

Since cellular systems are complex and often consist of multiple, interlocked feedback loops. Understanding of how the loops act together in giving rise to the system dynamics is absolutely crucial. Designs with interlinked positive and negative feedback loop have been shown to exhibit performance advantages over simple negative feedback loops, such as the ability to easily tune frequency of oscillators, improved robustness and reliability, even under noisy environments [22,27,50]. Multiple-negative-feedback-loop designs have also been shown to enhance system robustness and generates developmental constancy [8,27,47,51]. We obtained in this study a number of results which further our understanding into the dynamics of coupled-loop systems. We discuss these below.

**First**, coupled loops effectively enable oscillations at lower, more biologically plausible Hill coefficient value. For example, a four-species single loop Goodwin system requires the Hill coefficient (*n*_1_) to be at least 4 for oscillations. Its variant design with the loop reallocated to impose on the second pathway step requires the Hill coefficient (*n*_2_) to be at least 8 for oscillations. However, a system with both of these loops in effect can achieve oscillations at practically any Hill coefficient value for one loop, given proper choice of the Hill coefficient for the other loop. Oscillations, therefore, are possible at more biologically plausible Hill coefficients, for example at (*n*_1_, *n*_2_) = (3, 3) or (2, 4).

Reduction of the Hill coefficient for oscillations is often only suggested via pathway lengthening by previous studies. In this study, loops coupling and end-product utilisation (discussed above) were shown as the two additional mechanisms where this reduction can be obtained without increasing the number of system variables.

**Secondly**, coupled-loop systems were also shown to exhibit much greater complexity and more diverse behaviours compared to their single-loop counterparts. We showed that, by having just two loops performing cooperatively, the four-species system demonstrates a rich diversity of dynamical characteristics. For example, we detected a total of up to13 different bifurcation patterns between the feedback strengths. This enhancement in behavioral complexity and diversity might be the reason why evolution has driven some systems to acquire multiple feedback regulations as it will increase the chance of organisms' survival when facing fluctuating environments.

**Thirdly, **we found that different combinations of feedback strengths of individual loops give rise to different dynamical regimes. For three species with double loops acting on the first and second steps, stability is most probable when a weak first loop is coupled with a strong second loop. Oscillations, on the other hand, are most likely if a weak second loop is coupled with a strong first loop. If oscillations are to be obtained with a strong first loop, the second loop must also be significantly strong.

**Fourthly**, we found a threshold strength for the first loop. If the loop is weaker than this threshold, the system is always stable regardless of the strength of the second loop. This threshold strength turns out to be independent of the second loop's specification (its strength and cooperativity level). On the contrary, at any strength of the second loop, stable as well as oscillatory dynamics are obtainable given a proper choice of the first loop's strength. By further considering the coupled-loop system consisting of the loops on the first and the third reaction step, we discovered that the location of the additional loop has no influence on the threshold strength of the first feedback loop.

**Finally**, examining the system with all three loops in action showed that incorporating the extra third loop always enhances system stability. The likelihood of having oscillatory behaviour is directly determined by the loops' strength: stronger loops always result in smaller oscillatory region.

We demonstrate the practicality of our analysis by including a brief investigation of two example systems: the Hes1 oscillator and the Tryptophan operon system. The former system represents a single-loop system while the latter represents one with multiple negative feedback loops coupled together. Because of the abundant number of biological systems regulated by negative feedback loops (and many can be represented under simplifying assumptions by one of the motifs considered here) the methods developed in this study may prove useful in gaining better understanding of their dynamical behaviours.

## Authors' contributions

LKN devised the work, carried out the mathematical research and implemented the numerical simulations with guidance from DK. LKN and DK wrote the paper. Both authors have read and approved the final version of the manuscript.

## Supplementary Material

Additional file 1 **Supplementary Information**. This file consists of three parts. Section 1 presents the mathematical derivations for the results involving the single-loop systems. Section 2 presents the mathematical derivations for the results involving the coupled-loop systems. Section 3 presents the mathematical derivations for the results involving the system with endproduct utilisation. Section 4 gives the explicit expressions of the coefficients of functions *f *and *g *discussed in the main text, and some intermediate derivation steps. The supplementary figure S1 is given in section 5. Click here for file
