# Supplementary material for: On the functional diversity of dynamical behaviour in genetic and metabolic feedback systems
Source: BMC Syst Biol. 2009 May 11;3:51. doi: 10.1186/1752-0509-3-51 (PMC2705352; doi:10.1186/1752-0509-3-51)
Supplement: Additional file 1 — Supplementary Information. This file consists of three parts. Section 1 presents the mathematical derivations for the results involving the single-loop systems. Section 2 presents the mathematical derivations for the results involving the coupled-loop systems. Section 3 presents the mathematical derivations for the results involving the system with endproduct utilisation. Section 4 gives the explicit expressions of the coefficients of functions f and g discussed in the main text, and some intermediate derivation steps. The supplementary figure S1 is given in section 5. [file 1752-0509-3-51-S1.doc]

Supplementary material for the paper

On the functional diversity of dynamical behaviour in genetic and metabolic feedback systems

**Lan K. Nguyen^1^, Don Kulasiri ^1§^**

Additional file 1:

**Supplementary Material**

This file contains the material which is not given in the paper due to the space limitations. This file consists of three parts. Section 1 presents the mathematical derivations for the results involving the single-loop systems. Section 2 presents the mathematical derivations for the results involving the coupled-loop systems. Section 3 presents the mathematical derivations for the results involving the system with endproduct utilisation. Section 4 gives the explicit expressions of the coefficients of functions *f* and *g* discussed in the main text, and some intermediate derivation steps. The supplementary figure S1 is given in section 5.

1. Single-Loop Systems

The system’s local stability can be known from the eigenvalues of the system characteristic polynomial. For the three-species system with all three negative feedback loops, the corresponding characteristic polynomial is a cubic given by

$f(\lambda)=\lambda^{3}+\alpha_{1}\lambda^{2}+\alpha_{2}\lambda+\alpha_{3}$, (1)

with coefficients

$\begin{matrix} \alpha_{1}=k_{\text{d}1}+k_{\text{d}2}+k_{\text{d}3}+k_{\text{d}3}M_{3}n_{3} \\ \alpha_{2}=k_{\text{d}1}k_{\text{d}2}+k_{\text{d}1}k_{\text{d}3}+k_{\text{d}2}k_{\text{d}3}(1+M_{2}n_{2})+(k_{\text{d}1}+k_{\text{d}2})k_{\text{d}3}M_{3}n_{3} \\ \alpha_{3}=k_{\text{d}1}k_{\text{d}2}k_{\text{d}3}(1+M_{1}n_{1}+M_{2}n_{2}+M_{3}n_{3}) \end{matrix}$ (2)

where

$M_{1}=\frac{\bar{x}_{3}^{n_{1}}}{K_{1}^{n_{1}}+\bar{x}_{3}^{n_{1}}}$,$M_{2}=\frac{\bar{x}_{3}^{n_{2}}}{K_{2}^{n_{2}}+\bar{x}_{3}^{n_{2}}}$,$M_{3}=\frac{\bar{x}_{3}^{n_{3}}}{K_{3}^{n_{3}}+\bar{x}_{3}^{n_{3}}}$. (3)

According to the Routh-Hurwitz criteria, the system’s stability condition is

$\alpha_{1}>0, \alpha_{2}>0$and$\alpha_{1}\alpha_{2}>\alpha_{3}$ (4)

Since *α*_1_ and *α*_2_ are obviously positive, the stability condition is therefore reduced to

$\alpha_{1}\alpha_{2}>\alpha_{3}$ (5)

The steady-state (equilibrium) values of the system variables can be determined by setting the right hand sides of the system model equations to zeros. This means

$\begin{matrix} k_{1}\frac{K_{1}^{n_{1}}}{K_{1}^{n_{1}}+\bar{x}_{3}^{n_{1}}}-k_{\text{d}1}\bar{x}_{1}=0 \\ k_{2}\bar{x}_{1}\frac{K_{2}^{n_{2}}}{K_{2}^{n_{2}}+\bar{x}_{3}^{n_{2}}}-k_{\text{d2}}\bar{x}_{2}=0 \\ k_{3}\bar{x}_{2}\frac{K_{3}^{n_{3}}}{K_{3}^{n_{3}}+\bar{x}_{3}^{n_{3}}}-k_{\text{d3}}\bar{x}_{3}=0 \end{matrix}$ (6)

Working from the bottom of equations (6) up we have

$\begin{matrix} \bar{x}_{3}=\frac{k_{3}}{k_{\text{d3}}}\bar{x}_{2}\frac{K_{3}^{n_{3}}}{K_{3}^{n_{3}}+\bar{x}_{3}^{n_{3}}}=\frac{k_{3}}{k_{\text{d3}}}\left(\frac{k_{2}}{k_{\text{d2}}}\bar{x}_{1}\frac{K_{2}^{n_{2}}}{K_{2}^{n_{2}}+\bar{x}_{3}^{n_{2}}}\right)\frac{K_{3}^{n_{3}}}{K_{3}^{n_{3}}+\bar{x}_{3}^{n_{3}}} \\ =\frac{k_{3}}{k_{\text{d3}}}\left(\frac{k_{2}}{k_{\text{d2}}}\left(\frac{k_{1}}{k_{\text{d}1}}\frac{K_{1}^{n_{1}}}{K_{1}^{n_{1}}+\bar{x}_{3}^{n_{1}}}\right)\frac{K_{2}^{n_{2}}}{K_{2}^{n_{2}}+\bar{x}_{3}^{n_{2}}}\right)\frac{K_{3}^{n_{3}}}{K_{3}^{n_{3}}+\bar{x}_{3}^{n_{3}}} \\ =\frac{k_{1}k_{2}k_{3}}{k_{\text{d}1}k_{\text{d2}}k_{\text{d3}}}\frac{K_{1}^{n_{1}}}{K_{1}^{n_{1}}+\bar{x}_{3}^{n_{1}}}\frac{K_{2}^{n_{2}}}{K_{2}^{n_{2}}+\bar{x}_{3}^{n_{2}}}\frac{K_{3}^{n_{3}}}{K_{3}^{n_{3}}+\bar{x}_{3}^{n_{3}}} \end{matrix}$ (7)

Substitute (3) into (7) we have the equilibrium equation reduced to

$(1-M_{1})(1-M_{2})(1-M_{3})=A\bar{x}_{3}$, with $A=\frac{k_{\text{d}1}k_{\text{d2}}k_{\text{d3}}}{k_{1}k_{2}k_{3}}$ (8)

1.1. System$L_{3}^{1}$

1.1.1 Stability Condition

In this case we have *M*_2_=*M*_3_=0, equation (5) now becomes after substitution from (2):

$(k_{\text{d}1}+k_{\text{d}2}+k_{\text{d}3})(k_{\text{d}1}k_{\text{d}2}+k_{\text{d}1}k_{\text{d}3}+k_{\text{d}2}k_{\text{d}3})>k_{\text{d}1}k_{\text{d}2}k_{\text{d}3}(1+M_{1}n_{1})$,

or

$M_{1}n_{1}<\frac{(k_{\text{d}1}+k_{\text{d2}})(k_{\text{d2}}+k_{\text{d3}})(k_{\text{d3}}+k_{\text{d1}})}{k_{\text{d}1}k_{\text{d2}}k_{\text{d3}}}$. (9)

Denote the right hand side of (9) by *B* then the stability condition becomes

$M_{1}<\frac{B}{n_{1}}$. (10)

1.1.2 Critical n_1_

Applying the Cauchy-Schwartz inequality for 2 variables we have:

$$k_{\text{d}1}+k_{\text{d}2}\geq2\sqrt{k_{\text{d}1}k_{\text{d}2}}$$

$$k_{\text{d2}}+k_{\text{d3}}\geq2\sqrt{k_{\text{d2}}k_{\text{d3}}}$$

$$k_{\text{d3}}+k_{\text{d1}}\geq2\sqrt{k_{\text{d3}}k_{\text{d1}}}$$

Multiply these inequalities together we obtain *B* ≥ 8. Equality occurs when *k*_d1_= *k*_d2_= *k*_d3_. Since *M*_1_<1 always, condition (6) is always satisfied if *n*_1_ ≤ 8; in this case the system is always stable regardless of the other parameters’ values.

1.1.3 Choice of Parameters for Oscillatory System

Because *B* is a continuous function of *k*_d1_, *k*_d2_, *k*_d3_ with its minimum is 8, *B* therefore can take any values that is greater than 8 with some *k*_d1_, *k*_d2_, *k*_d3_. For example, we choose *k*_d1_= *k***k*_d2_=*k***k_d3_* we have

$B=\frac{2(1+k)^{2}}{k}\to\infty$ as $k\to\infty$.

If *n*_1_>8, this means that there exists an indefinite number of parameter sets {*k*_d1_, *k*_d2_, *k*_d3_} that makes *B*<*n*_1_ and so$B/n_{1}<1$. On the other hand, we can always choose *K*_1_ large enough to make *M*_1_ closer to 1 and exceeds$B/n_{1}$. This violates the stability condition (10) and hence destabilises the system. Because the choice of *k*_d1_, *k*_d2_, *k*_d3_ and *K*_1_ are independent, the choice of a proper parameter set is thus justified.

1.1.4 Derivation of the threshold *K*_1thesh_

Here we derive the stability condition for the inverse feedback strength indicator *K*_1_ as function of the remaining model parameters.

Substitute *M*_2_=*M*_3_=0 into equation (8), the equilibrium equation for system $L_{3}^{1}$ reads

$1-M_{1}=A\bar{x}_{3}$. (11)

Since$M_{1}=\frac{\bar{x}_{3}^{n_{1}}}{K_{1}^{n_{1}}+\bar{x}_{3}^{n_{1}}}$, we have$\bar{x}_{3}=K_{1}\left(\frac{M_{1}}{1-M_{1}}\right)^{\frac{1}{n_{1}}}$. Substitute (11) into this we obtain

$1-M_{1}=AK_{1}\left(\frac{M_{1}}{1-M_{1}}\right)^{\frac{1}{n_{1}}}$,

or

$K_{1}=\frac{1-M_{1}}{A}\left(\frac{1-M_{1}}{M_{1}}\right)^{\frac{1}{n_{1}}}=fun(M_{1})$. (12)

Equation (12) shows that *K*_1_ is a function of *M*_1_. This function is strictly decreasing with respect to *M*_1_ due to (note that 0<*M*_1_<1)

$\frac{\delta fun(M_{1})}{\delta M_{1}}=-\frac{1}{A}\left(\frac{1-M_{1}}{M_{1}}\right)^{\frac{1}{n_{1}}}\left(1+\frac{1}{M_{1}n_{1}}\right)<0$.

As a result, the stability condition (10) is equivalent to:

$K_{1}>fun\left(\frac{B}{n_{1}}\right)=\frac{(n_{1}-B)^{1+1/n_{1}}}{An_{1}B^{1/n_{1}}}$. (13)

Denote the right hand side of (13) *K*_1thresh_, the *K*_1_’s threshold value, it acts as a function of the Hill coefficient *n*_1_ and two compound variables *A* and *B*. This threshold value of *K*_1_ bifurcates the system between the stable and oscillatory dynamics.

1.1.5 Critical K_1_

Here we find the analytical form of the critical *K*_1_ discussed in the text. We will show that this value is 1/*A* for the range of *n*_1_ of interest.
Put $k=\frac{n_{1}}{B}$ then $n_{1}=k B$. Note that we only need to consider *k* ≥ 1 (so that *n*_1_  *B*). Condition (13) now transforms to

$K_{1\text{thresh}}=\frac{1}{A}\left(\frac{k-1}{k}(k-1)^{\frac{1}{kB}}\right)$. (14)

- If 1≤ *k* < 2 then trivially$K_{1\text{thresh}}<\frac{1}{A}$ (16)
- If *k* ≥ 2, we have (since *B* ≥ 8) $\frac{k-1}{k}(k-1)^{\frac{1}{kB}}<\frac{k-1}{k}(k-1)^{\frac{1}{8k}}$. This function is < 1 for *k* ≤ 1000, therefore $K_{1\text{thresh}}<\frac{1}{A}$ (17)

for *k* up to 1000 or *n*_1_ up to 8000 (this well includes the plausible range of *n*_1_).

Moreover, since

$\lim_{k\to\infty} \left(\frac{k-1}{k}(k-1)^{\frac{1}{kB}}\right)=1$ then $\lim_{k\to\infty} K_{1\text{thresh}}=\frac{1}{A}$, (18)

combining (16-18), we can conclude that the critical value for *K*_1_ is$\frac{1}{A}$.

1.1.6 Effects of the Degradation rate on B

Here, we will investigate conditions under which *B* is low or high.

Put$a=kd_{2}/kd_{1}$, $b=kd_{3}/kd_{2}$and $c=kd_{1}/kd_{3}$we obtain

$B=(a+1)(b+1)(c+1)$ with$abc=1$.

*B* is large when either *a*, *b*, or *c* is large; meaning among the degradation parameters, one is many folds greater than another ($kd_{i}\gg kd_{j}$with $i,j\in\left\{1,2,3\right\}$). On the other hand, *B* is low when$kd_{1}\approx kd_{2}\approx kd_{3}$.

1.2. System$L_{4}^{1}$

1.2.1 Critical n_1_ (Proving B ≥ 4)

*B* in this case has the following form

$B=\frac{(k_{\text{d}1}+k_{\text{d}2})(k_{\text{d}1}+k_{\text{d}3})(k_{\text{d}2}+k_{\text{d}3})(k_{\text{d}1}+k_{\text{d4}})(k_{\text{d}2}+k_{\text{d4}})(k_{\text{d}3}+k_{\text{d4}})}{k_{\text{d}1}k_{\text{d}2}k_{\text{d}3}k_{\text{d4}}(k_{\text{d}1}+k_{\text{d}2}+k_{\text{d}3}+k_{\text{d4}})^{2}}$ (19)

Here we show that *B* ≥ 4 for all positive degradation rates.

Note that

$\begin{matrix} (k_{\text{d1}}+k_{\text{d2}})(k_{\text{d2}}+k_{\text{d3}})(k_{\text{d3}}+k_{\text{d4}})(k_{\text{d4}}+k_{\text{d1}})-(k_{\text{d1}}+k_{\text{d2}}+k_{\text{d3}}+k_{\text{d4}})(k_{\text{d1}}k_{\text{d2}}k_{\text{d3}}+k_{\text{d1}}k_{\text{d2}}kd_{4}+k_{\text{d1}}k_{\text{d3}}kd_{4}+k_{\text{d2}}k_{\text{d3}}k_{\text{d4}}) \\ =(k_{\text{d1}}k_{\text{d3}}-k_{\text{d2}}k_{\text{d4}})^{2}\geq0, \end{matrix}$which leads to

$$\begin{matrix} (k_{\text{d1}}+k_{\text{d2}})(k_{\text{d2}}+k_{\text{d3}})(k_{\text{d3}}+k_{\text{d4}})(k_{\text{d4}}+k_{\text{d1}})(k_{\text{d1}}+k_{\text{d3}})(k_{\text{d2}}+k_{\text{d4}})\geq\\ \geq(k_{\text{d1}}+k_{\text{d2}}+k_{\text{d3}}+k_{\text{d4}})(k_{\text{d1}}k_{\text{d2}}k_{\text{d3}}+k_{\text{d1}}k_{\text{d2}}k_{\text{d4}}+k_{\text{d1}}k_{\text{d3}}k_{\text{d4}}+k_{\text{d2}}k_{\text{d3}}k_{\text{d4}})(k_{\text{d1}}+k_{\text{d3}})(k_{\text{d2}}+k_{\text{d4}})= \\ =(k_{\text{d1}}+k_{\text{d2}}+k_{\text{d3}}+k_{\text{d4}})\left(4k_{\text{d1}}k_{\text{d2}}k_{\text{d3}}k_{\text{d4}}(k_{\text{d1}}+k_{\text{d2}}+k_{\text{d3}}+k_{\text{d4}})+(k_{\text{d1}}-k_{\text{d3}})^{2}(k_{\text{d2}}+k_{\text{d4}})k_{\text{d2}}k_{\text{d4}}+(k_{\text{d2}}-k_{\text{d4}})^{2}(k_{\text{d1}}+k_{\text{d3}})k_{\text{d1}}k_{\text{d3}}\right)\geq\\ \geq4k_{\text{d1}}k_{\text{d2}}k_{\text{d3}}k_{\text{d4}}(k_{\text{d1}}+k_{\text{d2}}+k_{\text{d3}}+k_{\text{d4}})^{2} \end{matrix}$$

This means$B\geq4$and equality occurs when *k*_d1_= *k*_d2_= *k*_d3_= *k*_d4_.

1.2.2 Choice of Parameters for Oscillatory System

Similar to section 1.1.2

2. Coupled-Loop Systems

System$L_{3}^{1,2}$ - Derivation of the Critical *K*_1_

To derive this *K*_1crit_, we express the *SC* in terms of *M*_2_ and perform analysis on the *M*_1_-*M*_2_ coordinate:

***M*_2_(*M*_1_) > *g*(*M*_1_)**,

with

,

and

$g(M_{1})=\frac{b_{1}M_{1}}{a_{1}}-\frac{a_{0}}{a_{1}}$.

At *K*_1_=*K*_1crit_: *M*_2_(*M*_1_) = *g*(*M*_1_) = 0. This means $M_{1}=\frac{a_{0}}{b_{1}}$, so at *K*_1crit_ which gives us

.

*K*_1crit_ is a function of only *n*_1_, production and degradation rates.

3. Incorporation of End-product Utilisation in the Model

Here we derive the results for the case with modified degradation rate for the inhibitor species.

Model equations

$\begin{matrix} x'_{1}=k_{1}\frac{K_{1}^{n_{1}}}{K_{1}^{n_{1}}+x_{4}^{n_{1}}}-k_{\text{d}1}x_{1} \\ x'_{2}=k_{2}x_{1}-k_{\text{d}2}x_{2} \\ x'_{3}=k_{3}x_{2}-k_{\text{d}3}x_{3} \\ x'_{4}=k_{4}x_{3}-k_{\text{d4}}x_{4}-g \end{matrix}$ (20)

Steady-state (equilibrium) values of the system variables can be determined by setting the right hand sides of (20) to zeros

$\begin{matrix} k_{1}\frac{K_{1}^{n_{1}}}{K_{1}^{n_{1}}+\bar{x}_{4}^{n_{1}}}-k_{\text{d}1}\bar{x}_{1}=0 \\ k_{2}\bar{x}_{1}-k_{\text{d}2}\bar{x}_{2} =0 \\ k_{3}\bar{x}_{2}-k_{\text{d}3}\bar{x}_{3} =0 \\ k_{4}\bar{x}_{3}-k_{\text{d4}}\bar{x}_{4}-g =0 \end{matrix}$ (21)

This can be reduced to

$1-M_{1}=A\bar{x}_{4}+G$, with$A=\frac{k_{\text{d}1}k_{\text{d2}}k_{\text{d3}}k_{\text{d4}}}{k_{1}k_{2}k_{3}k_{4}}$, $G=\frac{Ag}{k_{\text{d4}}}$ (22)

It is important to note here that for (22) to make sense, we must have

$G<1$ or $g<\frac{k_{\text{d4}}}{A}=\frac{k_{1}k_{2}k_{3}k_{4}}{k_{\text{d}1}k_{\text{d2}}k_{\text{d3}}}$ (23)

Now, we set up the stability condition based on the Routh-Hurwitz criteria. The system’s characteristic polynomial is a 4^th^-order one and has the following form

$f(\lambda)=\lambda^{4}+\alpha_{1}\lambda^{3}+\alpha_{2}\lambda^{2}+\alpha_{3}\lambda+\alpha_{4}$, (24)

with the coefficients

$\begin{matrix} \alpha_{1}=k_{\text{d}1}+k_{\text{d}2}+k_{\text{d}3}+k_{\text{d}4} \\ \alpha_{2}=k_{\text{d}1}k_{\text{d}2}+k_{\text{d}1}k_{\text{d}3}+k_{\text{d}2}k_{\text{d}3}+k_{\text{d}1}k_{\text{d}4}+k_{\text{d}2}k_{\text{d}4}+k_{\text{d}3}k_{\text{d}4} \\ \alpha_{3}=k_{\text{d}1}k_{\text{d}2}k_{\text{d}3}+k_{\text{d}1}k_{\text{d}2}k_{\text{d4}}+k_{\text{d}1}k_{\text{d3}}k_{\text{d4}}+k_{\text{d2}}k_{\text{d3}}k_{\text{d4}} \\ \alpha_{4}=k_{\text{d}1}k_{\text{d}2}k_{\text{d}3}k_{\text{d4}}\left(1+\frac{(1-M_{1})M_{1}n_{1}}{A\bar{x}_{4}}\right) \end{matrix}$. (25)

According to the Routh-Hurwitz criteria, the system’s stability condition is

$\alpha_{1}>0, \alpha_{2}>0$,$\alpha_{1}\alpha_{2}>\alpha_{3}$ and $(\alpha_{1}\alpha_{2}-\alpha_{3})\alpha_{3}-\alpha_{1}\alpha_{4}>0$. (26)

It is easy to see that (26) is equivalent to the last condition

$(\alpha_{1}\alpha_{2}-\alpha_{3})\alpha_{3}-\alpha_{1}\alpha_{4}>0$ (27)

Substitute (22) and (25) into (27), it now becomes

$\frac{(1-M_{1})M_{1}}{1-M_{1}-G}<\frac{B}{n_{1}}$, (28)

with $B=\frac{(k_{\text{d}1}+k_{\text{d}2})(k_{\text{d}1}+k_{\text{d}3})(k_{\text{d}2}+k_{\text{d}3})(k_{\text{d}1}+k_{\text{d4}})(k_{\text{d}2}+k_{\text{d4}})(k_{\text{d}3}+k_{\text{d4}})}{k_{\text{d}1}k_{\text{d}2}k_{\text{d}3}k_{\text{d4}}(k_{\text{d}1}+k_{\text{d}2}+k_{\text{d}3}+k_{\text{d4}})^{2}}$.

We solve (28) and obtain

$M_{1}<\frac{1}{2}\left(1+\frac{B-\sqrt{(B-n_{1})^{2}+4BGn_{1}}}{n_{1}}\right)=B_{g}$. (29)

It is easy to check 0<*B*_g_<1 for all positive *B*, *n*_1_ and *G*.

Since$M_{1}=\frac{\bar{x}_{4}^{n_{1}}}{K_{1}^{n_{1}}+\bar{x}_{4}^{n_{1}}}$, we have$\bar{x}_{4}=K_{1}\left(\frac{M_{1}}{1-M_{1}}\right)^{\frac{1}{n_{1}}}$. Substitute (22) into this we obtain

$1-M_{1}=AK_{1}\left(\frac{M_{1}}{1-M_{1}}\right)^{\frac{1}{n_{1}}}+\frac{Ag}{k_{\text{d4}}}$.

And so equivalently, we have

$K_{1}=\left(\frac{1-M_{1}-G}{A}\right)\left(\frac{1-M_{1}}{M_{1}}\right)^{\frac{1}{n_{1}}}=fun(M_{1})$ as a function of *M*_1_.

Since

$$\frac{\delta fun(M_{1})}{\delta M_{1}}=-\frac{1}{A}\left(\frac{1-M_{1}}{M_{1}}\right)^{\frac{1}{n_{1}}}\left(1+\frac{1}{M_{1}n_{1}}\right)\left(\frac{1-M_{1}-G}{1-M_{1}}\right)<0$$

then *K*_1_ is strictly decreasing with *M*_1_, this combined with (D.10) means the stability condition is equivalent to

$K_{1}>fun\left(B_{g}\right)=\left(\frac{1-B_{g}-G}{A}\right)\left(\frac{1-B_{g}}{B_{g}}\right)^{\frac{1}{n_{1}}}$.

If *G*=0 (*g*=0), *B*_g_ is reduced to $\frac{B}{n_{1}}$ for $n_{1}>B$. This is consistent with the results from the previous section.

4. Intermediate steps of mathematical derivation

4.1. Positive derivatives

$\frac{\partial}{\partial M_{2}}\left(\frac{M_{2}}{1-M_{2}}\right)=\frac{1}{(1-M_{2})^{2}}>0$, and

for values of *M*_2_ between 0 and 1.

4.2. Coefficients *a*_0_, *a*_1_ and *b*_1_ for system $L_{3}^{1,2}$

$a_{1}=kd_{2}kd_{3}(kd_{2}+kd_{3})n_{2}$, $b_{1}=kd_{1}kd_{2}kd_{3}n_{1}$ and$a_{0}=(kd_{1}+kd_{2})(kd_{2}+kd_{3})(kd_{3}+kd_{1})$.

4.3. Coefficients *a*_0_, *a*_1_, *a*_2_ and *b*_1_ for system $L_{3}^{1,3}$

$\begin{matrix} a_{2}=(k_{\text{d}1}+k_{\text{d}2})k_{\text{d}3}^{2}n_{3}^{2} \\ a_{1}=(k_{\text{d}1}+k_{\text{d}2})k_{\text{d}3}(k_{\text{d}1}+k_{\text{d}2}+2k_{\text{d}3})n_{3} \\ b_{1}=k_{\text{d}1}k_{\text{d}2}k_{\text{d}3}n_{1} \\ a_{0}=(k_{\text{d}1}+k_{\text{d}2})(k_{\text{d}2}+k_{\text{d}3})(k_{\text{d}3}+k_{\text{d}1}) \end{matrix}$.

4.4. Functions$f(M_{2})$and $g(M_{2})$for system $L_{3}^{1,2,3}$

,$g(M_{2})=\frac{a_{3}M_{3}^{2}+a_{2}M_{3}+a_{1}M_{2}+c_{1}M_{2}M_{3}+a_{0}}{b_{1}}$,

and

.

The coefficients are: $a_{0}=(k_{\text{d}1}+k_{\text{d}2})(k_{\text{d}2}+k_{\text{d}3})(k_{\text{d}3}+k_{\text{d}1})$, $a_{1}=k_{\text{d2}}k_{\text{d3}}(k_{\text{d2}}+k_{\text{d3}})n_{2}$, $a_{2}=(k_{\text{d1}}+k_{\text{d2}})k_{\text{d3}}(k_{\text{d1}}+k_{\text{d2}}+2k_{\text{d3}})n_{3}$, $a_{3}=(k_{\text{d1}}+k_{\text{d2}})k_{\text{d}3}^{2}n_{3}$,$b_{1}=k_{\text{d1}}k_{\text{d2}}k_{\text{d3}}n_{1}$ and$c_{1}=k_{\text{d2}}k_{\text{d}3}^{2}n_{2}n_{3}$.

4.5. Coefficients *a*_0_, *a*_1_, *a*_2_ for system $L_{4}^{1,2}$

$$a_{0}=-(kd_{1}+kd_{2})(kd_{1}+kd_{3})(kd_{2}+kd_{3})(kd_{1}+kd_{4})(kd_{2}+kd_{4})(kd_{3}+kd_{4})$$

$a_{1}=kd_{2}kd_{3}kd_{4}\left(\begin{matrix} kd_{1}^{3}+kd_{1}^{2}(kd_{2}+kd_{3}+kd_{4})+kd_{1}(kd_{2}^{2}+kd_{3}^{2}+kd_{4}^{2})+kd_{1}(kd_{2}kd_{3}+kd_{3}kd_{4}+kd_{4}kd_{2}) \\ -(kd_{2}^{2}+kd_{3}kd_{4})(kd_{3}+kd_{4})-kd_{2}(kd_{3}^{2}+kd_{4}^{2}+kd_{3}kd_{4}) \end{matrix}\right)n_{2}$, $a_{2}=kd_{2}^{2}kd_{3}^{2}kd_{4}^{2}n_{2}^{2}$, and$b_{1}=kd_{1}kd_{2}kd_{3}kd_{4}(kd_{1}+kd_{2}+kd_{3}+kd_{4})^{2}n_{1}$.

5. Supplementary Figure


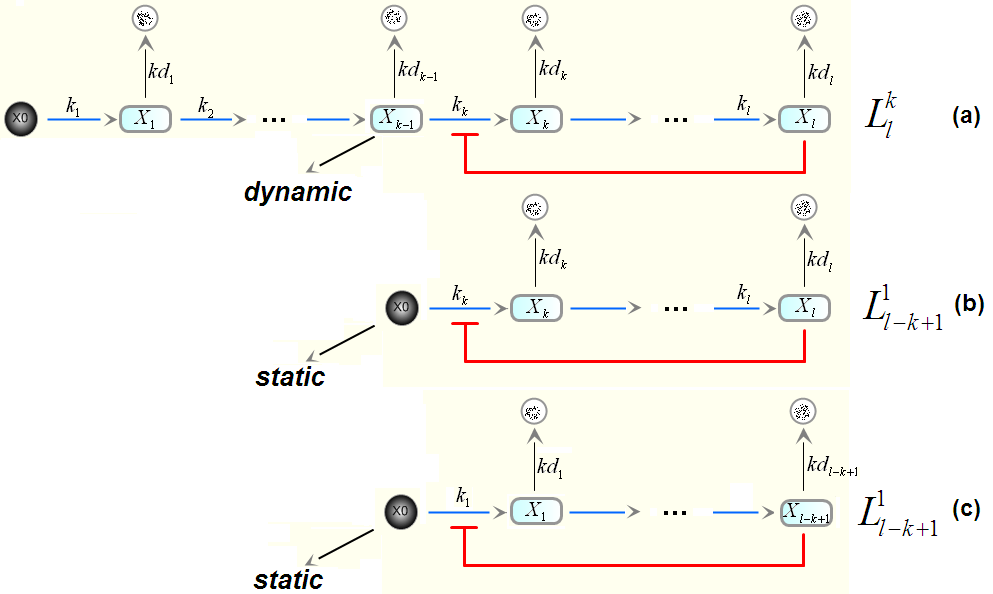


**Figure S1**

**Comparison of schematic topologies of $L_{l}^{k}$ and two versions of $L_{l-k+1}^{1}$.**
